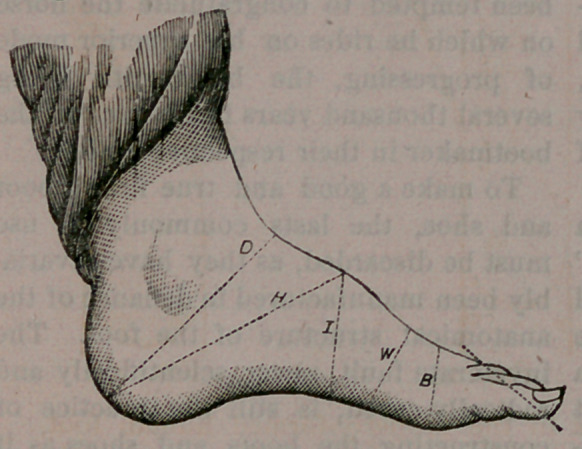# Miscellaneous

**Published:** 1874-10

**Authors:** 


### MISCELLANEOUS.

### THE CRABBE FAMILY.

Mr. Crabbe came home worn out by the labors of the day. It would have refreshed his spirit had he found his dressing-gown and slippers waiting by the fire, or even had some one brightened up and spoken pleasantly as he appeared. Nothing of the sort occurred. Mrs. Crabbe had indeed remembered the dressing-gown, but decided in her own mind that Mr. Crabbe was quite as able to wait upon himself as she was to wait upon him ; no one else had even thought of it. As for the pleasant greeting, not a word was uttered. The various members of the family looked up for an instant as the door opened, and seeing that it was "nobody but father," returned to their occupations. Mr. Crabbe stepped into the next room—to be sure it was but a step—and brought forth his evening dress, or undress, which he assumed amidst the continued stillness of the household.

"Where's the paper ?" he said, taking his own especial chair and preparing to make himself comfortable, since no other person showed any disposition to make him so.

There was a moment's delay in producing it. Young Rufus Crabbe, the oldest son, had been glancing over the news and thrown the sheet down carelessly in a corner. As it was damp from the press, this treatment had not improved its appearance. Mr. Crabbe uttered an ejaculation of impatience at the sight. If there were any thing he detested—and there were many things—it was a crumpled paper spread out in slovenly shapelessness, instead of being folded sharply and trimly down the middle. There was no comfort in reading such a rag. As he smoothed it out, endeavoring to reduce it to something like comeliness, a fresh cause of dissatisfaction became manifest.

"Humph !" he said, sniffing. "Cigars ! That boy's habits are intolerable."

Mrs. Crabbe flushed at this remark. "I don't think Rufus smokes more than other young men," she said.

"Other young men don't come into my house to do it, however," returned Mr. Crabbe ; "so that is not the question."

"I am sure he hardly ever smokes in the parlor," said Maria, his sister, taking up the defence in her turn.

"It is something he should never be allowed to do," replied the father, with emphasis.

"It is a pity, Mr. Crabbe," said his wife, "that you can not always be here to supervise our family arrangements. Perhaps you would be better suited than you seem to be at present."

"I have no doubt I should," he responded, cordially. Mrs. Crabbe was tempted to reply, but checked herself. The children had heard enough already. She continued her sewing with clouded brow and heightened color ; while Maria remarked to Gertrude in an under-tone that papa was dreadfully cross this evening, and Gertrude responded, "Yes, indeed ! Poor Rufe !"

Mr. Crabbe meanwhile perused his paper, denounced the policy of the opposite party, and rejoiced in every indication of the triumph of his own ; then pondered deeply the downward tendency of certain stocks, and questioned whether it were better to sell out at once and stand the loss, or hold on a while in hope of a rise. Amidst these musings the tea-bell rang.

"Where's Cecy ?" asked Mr. Crabbe, as they took their seats at table.

"She went to spend the afternoon with Marian Hammond," replied his wife.

"Oh !" said Mr. Crabbe. "That accounts."

No one asked for what. The information just satisfied a certain want in Mr. Crabbe's mind. Cecy was out. That accounted for his slippers being in the closet instead of by the fire ; that was the reason why the paper was not smoothly folded and airing on a chair-back ; that explained the general heaviness of the atmosphere.

Genial chit-chat was not the order of the day at the Crabbe table. It often chanced that some member of the family was brooding over injuries received at the hands of another, and so was indisposed for conversation, which cloud cast its portion of shadow over all. If there were no actual disturbance, each was usually too much occupied with his or her individual affairs to enter with much heartiness into topics of common interest. Cecy, indeed, sometimes brightened them up. Sometimes, also, the pervading moodiness overcame her, and she grew silent like the rest. To-night, in her absence, solemn stillness reigned unbroken till the entrance of Rufus, who threw himself into the vacant seat next his father.

"You're late, Sir!" said Mr. Crabbe, severely.

Rufus felt injured by the tone, as he was really not to blame.

"If you could give me some rule for finding people at home when they owe you money I might be earlier," he answered.

"Oh!" said Mr. Crabbe. "Where was it?"

"Davis's. As I was going down street I overtook young Lansing, and he told me that their firm paid in five thousand at the bank to-day to Davis's credit. I knew that now or never was our time, so I just turned on my track and took the street car up to his house. He wasn't in, and I had to wait an age; and when he came he was very offish, and not at all disposed to hand over. But I held on and let him see I knew he had the funds; and so, at last, by great good luck, I got it."

Mr. Crabbe was inwardly delighted by the news, for this account had been running on for a considerable time, and was held as more than doubtful. He was also gratified by the boy's promptness in attending to the matter. No hint of satisfaction, however, escaped his lips.

"What shape is it in?" he asked.

"Check—on the City Bank."

"Very well. You gave him a receipt, I suppose?"

"Yes, Sir. He wouldn't be very likely to let me go without one, I think."

"And the check is properly stamped?"

"You can look at it," replied Rufus, rather sullenly. "I believe I know enough of business to see that such things are attended to."

"Keep your temper, Sir," said Mr. Crabbe, "and remember to whom you are speaking."

Rufus bit his lips to hold back an angry answer. He had come in elated by his success, and conscious that he deserved some praise. A word of acknowledgment from his father, even a willingness to discuss the affair on equal terms, would have been enough for him. But Mr. Crabbe appeared to consider the subject closed, and finished his meal without further waste of speech.

As they went up stairs he relented a little. "Rufus," he said, "have that check sent in early to-morrow—as soon as the bank opens. You've managed the business so well thus far that it would be a pity to fail just in the end. I hardly thought we should ever see ourselves safe out of that scrape."

Rufus brightened at once. "I'll attend to it," he answered cheerfully. "Davis is a great scamp, isn't he, father?"

"H-m-m," said Mr. Crabbe, reflectively. "I can't just say about that. He appears to think the payment of his debts is money lost."

And all might now have gone smoothly had not something recalled to Mr. Crabbe's mind the injury he had suffered with reference to the evening paper. His son's recent service tempered the rebuke, but could not quite repress it.

"Rufus," he said, "when you have read the paper I shall be obliged if you will leave it in decent shape for those who come after you. And another thing. Tobacco is very offensive to me, particularly such cigars as you use. You have your own room, where you can smoke as much as you like, and the parlors must not be used for such a purpose. I hope you will remember this, and that I shall have no occasion to speak again upon the subject."

Mr. Crabbe might have stated his wishes in half a dozen different ways without arousing that quick spirit which abode in Rufus as in the rest of the family—always excepting Cecy. But there was a weight of dispassionate sternness in his tone that cut the youth deeply—and then the injurious reference to his cigars! Rufus bought them by the box of a "friend of his" down town, and considered that he obtained the choicest brands at a mere nominal price. To have his father, ignorant as an Esquimaux about such things, sneering at the quality of *his* cigars! It was a little too much. He left the room immediately, and the way in which he shut the door was not conciliatory. Shortly afterward the front door closed in a like energetic manner.

"Out again!" said Mr. Crabbe, as he heard it. "This business is rather overdone, I think. Our young masters will feel it a condescension, by-and-by, to board and lodge with us."

"I suppose he goes where he can have a little liberty," said Mrs. Crabbe, who had been inly exasperated at the slight notice taken of her boy's success and the after-piece of reproof. "It is not pleasant to be perpetually found fault with."

"Ah! unfortunate he should be so sensitive," remarked Mr. Crabbe, satirically.

"Unfortunate for him, certainly," answered the mother.

"Unfortunate for all his family," said Mr. Crabbe.

Mrs. Crabbe felt quite equal to a continuation of hostilities, but considered that the discussion had gone far enough, and allowed her husband the last word. Peace accordingly ensued for a time. The father, having finished his paper, took up a magazine; the mother and daughters went on with their needle-work.

"How cold it is!" said Gertrude, presently. "I do think this is the hardest room to keep warm in!"

"How can you say so?" returned Maria. "I'm comfortable enough, I'm sure."

"I dare say. I might be, too, if I had been sitting next the stove all the evening."

"There is no occasion for any one to suffer," remarked Mr. Crabbe. "Open the dampers and the room will soon be warm enough, I'll warrant."

Gertrude came around from her station at the back of the table and drew them open. Both Maria and the father were much nearer the stove; but then *they* were comfortable enough already. As it was Gertrude who desired the change, she was clearly the one to take the trouble of it—such little trouble as there was.

"Oh dear!" Maria speedily exclaimed. "The room is like a furnace. I'm sure you must be warm by this time, Gertrude."

"Indeed I am not. The heat hasn't got around yet."

"Do take my seat, then—it is fairly scorching here."

When matters had reached this pass Maria was quite willing to resign her place in favor of her sister.

"I never saw any one like you," she continued. "You are the *coldest* creature!"

"It isn't so, at all," replied Gertrude, injured by the accusation. "I am cold in a cold place, just as you would be yourself."

"There it is. Now *I* am just as comfortable here as I want to be."

"I wish you had changed with me a little sooner, then. And as for always being cold, I am no more given to complaining of it than you are. But you always think you are the standard, and if *you* feel warm enough every one ought to, and if you are chilly it's because it *is* chilly."

"Pray stop this wrangling," said Mr. Crabbe, looking up, "and let us have a little quiet."

Sympathy was immediately established between the sisters, united in a common hostility toward their parent. They drew nearer together, and agreed that papa wanted to keep the wrangling for his own especial privilege. Mrs. Crabbe, accustomed to these little breezes and by-plays, took no notice, but went on composedly with her sewing.

The evening was rather dull. There were no visitors; and after Mr. Crabbe's remonstrance the scene could hardly be enlivened by any little sparring. It was a welcome interruption, therefore, when, toward ten o'clock, the door-bell sounded and Cecy entered, fresh and glowing from the frosty air outside.

I can not say that Cecy Crabbe was handsome, or elegant, or in any way remarkable. Gertrude was quite the beauty of her circle, and Maria decidedly plain; they represented the extremes of the family, and Cecy its mean. She looked healthy and happy and kind-hearted; she had pretty brown eyes and pretty hair. Here I am afraid the catalogue of her charms must end.

"Had a pleasant visit, Cecy?" asked her father, laying down his book and smiling responsive to the smiling face.

"Oh yes; delightful!"

"Who was there?" said Gertrude.

"Only the family. I did not expect to meet any one, you know."

"You said it was so 'delightful,'" explained Gertrude, "I thought you must have had some one to relieve the Hammonds."

"I did not need any relief. Marian and I had ever so many things to talk about. And she is piecing a silk bed-quilt; the sweetest thing! I can't quite describe the figure, but I will cut out a block in paper to-morrow to show you. The colors shade into each other, and you have no idea how handsome it is. I am quite wild to make one. Mrs. Geer brought up the pattern with her from New York."

"She has come, has she?" asked Maria. "What does she seem like, Cecy? Stuck-up as ever?"

"I don't know—I did not see any thing of it. Was she so formerly?"

"'Was she,' indeed? Now, Cecy, you needn't pretend to so much innocence. There never was a girl in this city that put on such airs as Louise Hammond, and when she was engaged to that rich Mr. Geer she grew absolutely insufferable. Though what there was to plume herself on there I am sure *I* can't tell. A man old enough to be her father, and that she never would have looked at if it hadn't been for his money?"

"She seemed very pleasant to-night," said Cecy. "And, mother, you ought to see her twins. They are the sweetest little things!"

"As sweet as the silk bed-quilt?" inquired Mr. Grabbe.

"Ah, father, you're laughing at me. Yes, full as sweet, but in a different way. I don't expect you to appreciate patch-work, but I think you could not help admiring these babies. They are just a year old, and as lovely as if they were made to look at. The soft baby-complexion and such great dark eyes that fairly lit up their faces! I never saw such eyes," said Cecy, her own sparkling as she spoke.

"Look in the glass and you'll see them now, I guess," was Mr. Crabbe's comment.

"They are exactly alike," said Cecy, continuing her description. "I don't see how their own mother tells them apart. I tried, to-night, half a dozen times, and had to give up in the end and look at their necklaces to distinguish them."

"An exciting pastime," remarked Gertrude. "Cecy's delightful evening seems to have been spent in making out patch-work and trying to tell one baby from another!"

"Louise must have her hands full with the pair of them," said Mrs. Crabbe. "She pays pretty well for whatever privileges she has."

"Of course they are on her mind a good deal," replied Cecy, "but she has not much of the actual care. She keeps a nurse for each of them."

"Upon my word!" exclaimed Maria. "Two nurses! I wonder how the Hammonds relish that? Two smart city servants coming in upon them, with their small house and plain ways. I pity them!"

"They do not seem in need of pity," said Cecy, smiling. "They were in excellent spirits. Mrs. Geer, you know, is such a great character with them all."

"I dare say. Nothing like a rich marriage for raising your importance in every body's eyes."

"Suppose you try it for yourself," said Mr. Crabbe. "Perhaps then Louise Hammond's prosperity will not disturb you so much."

Maria was about to reply, but a glance from her mother checked her. She contented herself with looking injured and indignant the remainder of the evening.

"I brought home a famous recipe for muffins," said Cecy, hastening to introduce a new topic. "We had them for tea, and they were excellent. I mean to try them to-morrow, mamma."

"I wish you would," said Mr. Crabbe. "The bread to-night was simply dough."

"Betsy had to hurry that loaf in order to get in the meat for dinner," explained Mrs. Crabbe. "She very seldom has poor bread. But we can generally trust to your father to notice any little failure, and to inform us of it."

A quick shadow of discomfort crossed Cecy's face, and was gone again. "Father," she said, gayly, "can't you give me my revenge at backgammon to-night? There is time yet for a game or two."

"Mr. Crabbe assented, and the board was brought. No preliminary inquiries were needed; Cecy always played with the yellow men, and into the left-hand table. She was a dashing player—did not heed exposed points, and was indifferent to being taken up. Fortune favored her awhile, then veered shamelessly about to Mr. Crabbe.

"You're done for, Cecy," observed Rufus, who had come in and now stood overlooking the board.

"A gammon, I think, my dear," said her father.

"Don't be too certain," and at the words double-six rattled out, and released the four prisoners who had been held in durance vile through half a dozen throws.

"One or two more such strokes and you'll get in," said Rufus. But it was not to be. In her extremest need Cecy, like the Vicar of Wakefield, threw deuce-ace twice running. Mr. Crabbe's triumph was complete, and he closed the board in high good-humor, never dreaming but Cecy was as delighted with the pastime as he was himself.

A strong sense of *meum* and *tuum* pervades, as you might expect, the domestic system of the Crabbes. Each had his rights on which he stood, jealously keeping the ground. Some things were fully settled. Maria would no more have used Gertrude's thread or thimble without permission asked and given than she would have stolen fruit from the garden of a neighbor. Nor was such permission to be lightly sought. Gertrude would have said that there was no reason why Maria should not have thread of her own if she took the trouble to keep herself supplied; and as for her thimble, if she had a place for it, and put it there, she would have no need of borrowing. And Maria must have acknowledged the justice of these criticisms.

It was the same with their various duties. Each daughter had her allotted share, which she carefully fulfilled to its exact limits, not one hair's breadth over. She neither expected to receive nor to bestow assistance. Do you imagine that Maria dusted a picture-frame when Gertrude overlooked it during her week for clearing-up the parlors? Nay—not if it went undusted till the next Monday morning. She told Gertrude of it, with perchance a small sarcasm on her lack of nicety, and left her to repair the fault. There was no exchange of little kindly offices, lending of bows or collars, putting up hair and the like, so frequent in families less thoroughly regulated. Maria, indeed, had a gift for arranging trimmings or making up a head-dress; but Gertrude, if she sought her assistance in such matters, was heedful to tender an equivalent in other service. We may do Maria the justice to say that she would have demanded it had it not been offered. As in material things, so with moral. A spirit of indulgence was unthought of. No little failing was passed lightly over; no frac tion of unpleasant truth was ever withheld in defence to another's feelings.

One can hardly say whence this spirit was derived. I rather think from Mr. Crabbe, and that he was responsible, directly or indirectly, for the tone of the household. I know that in the first year of their wedded life Mrs. Crabbe was just as fondly watchful of her husband's comfort as any one could be; always ready to run up stairs for a clean pocket-handkerchief, or down stairs for a glass of water, did the occasion arise. Not that Mr. Crabbe was less able-bodied then than now, or less competent to supply his own needs, but that it was pleasant to render him such little service. She spent a good deal of time in preparing his favorite dishes, and wore the colors he preferred. But by-and-by the first child came, and Mr. Crabbe was not as kind and thoughtful as many husbands are. He grumbled when the baby cried at night, as if his wife were not kept awake as well. On any small domestic failure he animadverted freely; why should he not? he thought. It *was* a failure, it give him discomfort, and he should speak of it as often as it happened. Once or twice, when the presiding genius of the kitchen was away, he allowed his wife to make the fires, and did not himself arise till called to breakfast. The household labors were no part of his concerns, he told himself; he had his own business, and tired enough he got with it. It began to dawn on Mrs. Crabbe that all the little friendly offices came from her; that the glamour of courtship had passed away, and she was reduced to the common prosaic level of housekeeper and manager. It was not a pleasant discovery. If, like other women similarly enlightened, she said little about it, she thought much, and shed some bitter tears in secret even. One privilege at least she had; if she were no longer doted on she could, in turn, give up the foolishness of doting. Thus a portion of her character crusted over, and it came to pass that for many years Mr. Crabbe had been in her eyes the head and provider of the family, for whom, indeed, she had a certain regard, but whose faults she plainly saw, and whose shortcomings found in her no tenderness to excuse them.

You are not to suppose, however, that there was no semblance of family affection among the Crabbes. That were to do them great injustice. The children considered their father the very model of probity, intelligence, and sound judgment; their mother the best of mothers. It may be said for Mrs. Crabbe that she was a kind parent, and that her occasional sharpness was employed in behalf of others rather than herself. As for Mr. Crabbe, you would have insulted his understanding had you ventured to insinuate that any among the wives of his acquaintances possessed his own wife's skill in housekeeping, cookery, and general management, or was half as estimable a woman. His children were fully equal to other people's children, and in many things superior. Of Maria's music and Gertrude's beauty he was particularly proud, though all Maria knew of it was that her favorite variations were characterized as senseless rattle, without beginning, middle, or end; while Gertrude was painfully conscious that her mouth was much too wide to please his taste Rufus never suspected that his father thought him a fine, manly young fellow, quite a marvel, as boys went now-a-days. Cecy, without beauty, music, or special cleverness, was Mr. Crabbe's darling. Yet Cecy had her share of snubbing, too, if any thing were wrong.

In some respects the family management had its merits. It gave no scope for "shirking," whereby unpleasant duties are sometimes turned off on the more willing members of a household to an unjustifiable extent. It also prevented the wear and tear of mind often experienced by the one orderly sister, never able to count upon a clean collar or pocket-handkerchief, no matter what care she takes of them. Nor in this house was the father looked upon as an enemy from whom all possible tribute was to be wrung by various methods of wheedling, sulking, and surprises. Mr. Crabbe imparted of his substance as freely as his circumstances rendered prudent; wife and daughters accepted his estimate, had their allowance, and made the most of it. There were no underhand practices ; no bills run up at the milliner's and screwed out of the housekeeping money ; all was open and above-board.

But justice untempered by mercy is a hard rule for domestic life, or any other. The Crabbes were not unhappy. They sparred right and left, but, the combat once over, were on good terms again. Thus the knights of old, having given and received hard knocks, would sit down and feast together brotherly. But they might so easily have been a great deal happier; a little infusion of gentleness, of kind feeling, would have so softened and brightened their existence. So Cecy thought—and sighed. Sometimes when—rare occurrence—an evening had passed harmoniously, and they separated with perfect amity and good will on every hand, she wondered that they could not see it, that they did not think of it the next time any trifling provocation offered. She knew very well that if important service were required each was capable of a good deal of self-denial for another's sake. Why, then, could they not curb an impatient answer, repress a petty but vexatious fault-finding? She marveled that their religion did not lead them into greener paths, by stiller waters; for each and every individual of the family, save Rufus, was a member of the church in good and regular standing. But perhaps she was the first Crabbe who had ever dreamed of applying that sacred power to such profane and secular uses. Religion! that meant that you were not to lie, nor steal, nor swear, nor cheat; to defraud your servants of their wages, nor waste your own substance in riotous living; that you were to attend two services on Sunday, and, if extraordinarily devout, the church prayer-meeting on Thursday night. So far they were all agreed. In minor matters there was some difference of opinion. Mr. Crabbe considered that it also meant that you were not to dance; Mrs. Crabbe was not assured upon the subject, nor were Gertrude and Maria. Temptation was sometimes too strong for them, and on such occasions they came home with a painful heaviness at heart, uncertain whether they had sinned or innocently enjoyed themselves. Cecy could not see the harm, but chose to be on the safe side, and resolutely refrained, sure of thus pleasing best her earthly father at any rate. Religion further meant, with the Crabbes, the contribution of considerable sums to various authorized benevolent enterprises. Every Crabbe was a life-member in some Home or Foreign Mission or Bethel Society, the attestations whereof, handsomely framed, were distributed, by way of ornament, through the bedrooms of the house. It was not considered to forbid the gift of a pair of chickens to your washer-woman at Christmas, or kind offices to the poor in general, but these were held as quite secondary matters, and rather belonging to the barren realm of "works." In this religious system a good deal was taken for granted. It was assumed that all partakers of its benefits had passed from darkness into light; from death unto life; from the bondage of sin to the glorious liberty of children of God. Considering the momentous nature of these changes it is surprising how little effect they had, or were expected to have, upon the outward relations of those who had experienced them.

Time went on, bearing our family, harmonious or discordant, along on his resistless tide, till one morning Mr. Crabbe awoke, feeling very far from well. He was not the man to weakly yield to every passing ailment; therefore he arose, made an attempt at breakfast, and set out for business as usual. In the course of an hour or two he was brought home by Rufus in a hack, from which he was carried to his own room and thereafter treated as suited his condition.

In the Crabbe family bodily illness was a kind of sanctuary, so long as it lasted. It is true that complaining was looked upon with some suspicion, and that no encouragement was held out to any one to feel or fancy himself ailing without sufficient cause. But the point once established, the invalid was king. He was to be cared for, whatever else was done or left undone. He might revile his gruel or panada, grumble about his pillows, snap up ever so fiercely his zealous nurses; all was borne with patience, nay cheerfulness, and never for one instant laid up against him. He was sick—that excused and comprehended everything.

Mr. Crabbe in previous illness had availed himself of all these privileges; had gone, so to speak, the full length of his tether. But now he was strangely different; easily satisfied, seldom suggesting any fresh comfort or convenience, disposed rather to listless quiet than his usual caged restlessness. This of itself alarmed his wife. Then the physician began to come twice a day; there were anxious faces in the household, and dread forebodings of something too terrible to happen. Lower the patient sunk and lower. What was he thinking of in those long, silent days when he lay passively staring with blank eyes at the wall opposite? Or was the mind torpid while the body waked? Was he drawing, dull and unheeding, toward the solemn end? The hours passed on, measured by potion or nourishment; the children came and went, noiselessly, each filling in her turn the post of nurse. They looked with questioning awe at the pale face among the pillows; that awe which steals over us when the form, linked with all that was familiar, everyday, in our existence, begins to take to itself something of the remoteness of the great Hereafter; seems to belong *there* rather than here.

One night Mrs. Crabbe sat by the fire alone. It was late; all the house had long since gone to rest. Watchers in sick-rooms can recall these midnight vigils; the solemn stillness, through which eye and ear wait anxiously the slightest movement; the white bed with its pale, helpless tenant; and thought, busy every where, running back into the Past with longing, forward into the Future with trembling and with fear.

A slight movement called this watcher to the bedside. The invalid looked at her with large, solemn eyes, in which she saw a new expression—something different from the dull, hopeless weariness of suffering, something that told once more of consciousness and recognition. Her heart grew strangely tender at the sight. She leaned over and kissed his forehead. "Can I do anything for you, dear?" she asked.

"Not now. Stay by me, Mary. I want to speak to you."

She knelt at the side of the bed. "I am going to die," he said, with that wide, serious gaze still fixed upon her.

She did not shrink nor evade the issue by any commonplace of reassurance, but answered him with utter candor and eyes grave and steadfast as his own. "You are very ill, but we have not given up hope. We are in God's hands, and I trust He will spare you to us yet."

"I am going to die," the sick man repeated. "How good you have been to me, Mary; now and always. And I—I've been a bad husband to you."

"Oh, hush, James! Don't speak of such a thing. It is not so."

"Yes, Mary. And when I am gone you will always remember me—hard, and selfish, and exacting. It will be right that you should. And it might have been so different!"

Poor Mrs. Crabbe! These few words were enough. The accumulated ice of years melted before the glow of awakened feeling, and she saw in the poor, wasted form before her the love of her young days.

"You were not the only one," she said, amidst her tears. "I was to blame, too. I was too proud to complain—and how should you know?"

"I ought to have known. But you forgive me, Mary?"

"Yes, yes, my dear, if there is anything to forgive."

"We understand each other now, whatever happens," he said, with the shadow of a smile.

And then the nurse, dormant for the last few minutes, awoke again in Mrs. Crabbe. She remembered the danger of all agitation; she soothed and quieted the invalid, begging him to try to sleep; then sat down by the fire once more and cried, quietly, a perfect rain of tears. Not all unhappy ones, though she accused herself of many a fault, and sent up many a prayer for future guidance. If Mr. Crabbe had died then we may be certain that his wife's memories of him would have taken color from two periods: the happy fondness of their courtship, the tender gravity of his last hours. All the little rasping cares and worries would have passed out of mind; all that lay between those two extremes would have been brightened and softened by their influence.

Instead of dying he recovered; after a long, tedious convalescence, that gave ample room to test the strength of his convictions and the efficacy of his good resolves. I can not say that these last were never broken; that would be to proclaim that Mr. Crabbe was more than mortal. But he combated with marvelous success the risings of the old Adam within him. The children were ignorant of all that had passed through his mind in those days when he felt himself going down into the Valley of the Shadow, of the clear vision that revealed his own ungracious character, the self-reproach for his neglect of others' happiness, for the waste, or worse, of his own influence. But they could not be blind to the great change in his demeanor; he was so kind, so interested in all that happened to them, he so seldom spoke with the old crustiness that had seemed a portion of himself. As for Mrs. Crabbe, she found the sick-room a little Paradise; no need to stint now every kind office that her heart could dictate, for they no longer fell without appreciation or response. No maiden was ever more devoted to a lover than she to this oldish individual, in whom few eyes but her own would have discerned any charm. What a festal day it was when he first sat up, the dressing-gown wrapped carefully about his attenuated shape, his hair sprucely brushed by her own hands! With what content of heart she sat near him, busy with some light needle-work, that was never too urgent to be laid aside at any call for his comfort or amusement! What pleasant talks they had of their own earlier life and of the children's future!

So the days loitered on, and by-and-by Mr. Crabbe pronounced himself well enough to be about again. Business, so long a nullity, began to take its important place once more in the scheme of things. And here was fresh scope for any capacity of forbearance the invalid had acquired. All had perforce been left to Rufus; and Rufus had done well, his youth and inexperience considered; yet serious blunders had occurred. Mr. Crabbe had a hard struggle in his own mind, and came off victorious. He praised the general success, and passed lightly over the unlucky failures. Rufus blamed himself, when they were pointed out to him, far more severely than his father blamed him.

It soon became evident that Mr. Crabbe's restoration was by no means complete. The old spring, the old energy, were gone. People shook their heads in speaking of him, and said that illness was too much for him; he would never be the same man again. In some respects this was unfortunate. He had hoped in a year or two to have a better house, assume a better style of living, give his children a fairer start in life. All such hopes were reluctantly abandoned as the state of his health became manifest. He and his family must content themselves with such success as had already been achieved, and wait for more till Rufus, older and experienced, could infuse fresh life into business.

But the illness left other and enduring traces. Mr. Crabbe, kindly and companionable, was a different being from Mr. Crabbe, weary and impatient, who came home caring for nothing but his newspaper and his tea. His half-invalid state called for indulgence, for little cares, which every one was quick to render. In his presence small disputations were forborne—they annoyed father, it was understood, and father was not well; being thus forborne, they frequently passed out of mind, and left the air serene. If harshness and self-seeking had been contagious, good-will proved not less so. Cecy saw with delight a new spirit diffusing itself through the household; saw how demands were softened to requests, how quiet explanations took the place of curt replies, how sarcastic comments were repressed to silence. Gradually the stern rule of "every man for his own hand" relaxed; small kindnesses were interchanged, small self-denials practiced. Perhaps in those dark days when their father seemed verging toward the tomb the children's hearts had admonished them of a more excellent way than that in which they had walked hitherto; perhaps his changed example wrought the change in them. Whatever the cause, the effect was great and lasting. And thus the illness which clouded their fortune brightened their home, and proved the truest blessing to the whole Crabbe family.—*From Harper's Magazine.*

---

### HOW SHALL THE TEETH BE PRESERVED.

There is no inquiry more important than this. Life is unquestionably prolonged by a sound and perfect dental organization. In a thousand ways it is rendered comfortless and wretched by unsound and imperfect teeth.

The human teeth constitute the mill wherein all solid substances employed as food are comminuted, mixed, and reduced to a pasty, pulpy mass for the ready acceptance of the stomach. If the mill is in bad condition, the work will be imperfectly done, and the stomach—a very watchful and particular customer—will not fail to complain. So long as the mill is moderately fed with good material, and that material is reduced and prepared in a strictly workmanlike manner, the stomach is contented and happy, and performs its allotted task, year in and year out, with patient ease and never-ending satisfaction. But let the mill be supplied with improper substances, such as the stomach has not bargained for ; or let the supply be either greatly in excess or greatly below the stipulated amount ; or let the preparation be imperfect, and complaint will surely be made. The customer gives small heed to the cause of that unfair dealing which lessens his own comfort and his power for good. He simply utters his complaint and enforces it. The enforcement comes in the form of dyspepsia, debility, nervous disorders, and a low vital condition. It stands to reason, then, not only that the mill should be supplied with what its only customer demands—a customer who, when well-used, possesses a most unerring judgment—but also that the material supplied should be ground and mixed with conscientious fidelity.

Whatever of sickness may come to the owner of a good set of teeth, it is a fact which challenges denial, that perfect health cannot accompany imperfect teeth. The stomach cannot assimilate food improperly prepared. If from haste or bad teeth we swallow our food in a crude state, we may rest assured not only that our food will become unpalateable but that good digestion will not wait on appetite, nor health on both.

The mill, then, must be kept in order. We repeat what we have so often affirmed, that the chiefest necessity in the preservation of the teeth is cleanliness. Other measures are useful ; this is essential. How can this essential condition be most readily secured ? Unquestionably by the use of a firm substantial brush after eating. But the employment of the brush can be supplemented, with great advantage, by the use of a proper dentifrice. While no astringent, antiseptic, or aromatic substance should be allowed to take the place of the brush, a skilfully prepared combination of the best ingredients having these characteristics is of real service. Decay is practically solution. The acids generated in the mouth by the decomposition of adherent particles of food are the chief solvent. To remove these destructive particles is the office of the brush ; to neutralize the resulting acid is the business of the dentifrice.

In our search for an article fulfilling the needed conditions of a good dentifrice, we have tested nearly all the popular compounds, and have settled down on the preparation of the well-known chemist, George J. Wenck, which he puts up under the name of Eau Oraline. The prescription upon which it is based is by the well-known dentist, Dr. J. H. Haughwout. The fluid is evidently compounded of choice substances, selected with a view to strengthen the gums and the membranes connecting them with the teeth, and to impart an agreeable flavor to the mouth. We can conscientiously recommend its use, as we believe it to be calculated to accomplish much good. We assert, unhesitatingly, that if applied faithfully every night and morning with a good stiff brush, a sweet and healthy condition of the mouth and teeth may be maintained. It can be obtained through the wholesale agents, Messrs. Lord & Taylor, New York.

---

### TASTE IN DRESS.

Society judges men, to a certain extent, by their dress. It troubles itself little how humble your fare may be when you don't invite it to eat with you ; it allows you to live up any number of stairs if you don't ask it to climb them ; but for dress, in cut and freshness, it expects all men courting it to be equal. A certain amount of attention on this matter every one owes both to himself and the world. Beyond that, happy, mean solicitude on the subject becomes small and unmanly. * * *

I will venture to suggest a few special points to those who aim at a high-bred standard. Dressiness is to dress what staginess is to the stage. It defeats its own end. Follow the fashion, but at a respectful distance. Avoid all materials with a pattern, even for cravats and waistcoats. They might be called ugly, and that would be sad ; they might be called pretty, and that would be fatal. Whatever the color of your coat, never let it be lighter than your trousers. This does not apply to an overcoat. Anything like *fancy* in boots or shoes—as white stitching, buttons that don't really button, and so forth—would kill the otherwise most faultless get-up. Any article which looks "nice" on a tailor's show table, or in a shop window, must be distrusted. From Autumn till Spring, never show shirt-front or wear white waistcoats, except, of course, in evening dress. Cuffs must be positively made *on* the shirt, and collars are better the same, but this is not essential. A watch chain must represent much money. Diamonds can only be worn in a finger ring. Studs are only allowable in evening dress, except small, plain ivory or mother-of-pearl ones. Velvet collars can be worn only in Winter, and then but on frock, evening, or overcoats. Avoid the Parisian defect of letting your clothes look as if they were ironed on the person after being first glued to it ; neither must you have your things so loose as to make it appear that they have been thrown at you, and hung there. That which is quite suitable upon a very young and handsome man becomes *outre* if worn by one devoid of these advantages. All artifices employed for correcting the shortcomings of Nature are commendable, provided (as ladies, alas ! do not always remember when they make up their faces) they defy detection ; but otherwise the fraud only directs attention to the defect it was intended to hide. Even when one shoulder is a little higher than the other, experience shows that there is more lost than gained by padding up the falling side. This absurd custom is very prevalent. I met an extreme case in point last Thursday, and have not yet recovered from the shock. Hearken to what I had to endure from the ignorance or carelessness of a fellow-creature.

His hat said he was poor, and was contradicted flat by a diamond pin; while the shirt, though in the very grip of that jewel, boldly called out to the hat that it agreed with it. The gauzy cravat announced a sultry day, while the seal-skin waistcoat told me of mid-winter. A black velvet collar and patent-leather boots spoke of ceremonious visits to be paid; but the coat of gray tweed, and thick dog-skin gloves, laughingly exulted in their own announcement that they would be thrust out of any lady's drawing-room, and informed me, without any false shame, that a cock-fight was the probable destination of their wearer. The black trousers, calling my attention to the silk braid down their sides, darkly hinted at balls and parties.

In conclusion, avoid solecisms. Remember that every object which meets the eye is always saying something. A face in a moment of repose, a suit of clothes upon a man, or it matters not what example we take—everything, I say, is ever speaking to the eye of all observers; and note, when not speaking sense, speaking nonsense. I know nothing capable of talking such mute nonsense as the attire of an ill-dressed man, or which is therefore so opposed to what I started by speaking about—common sense.—*Baldwin's Monthly.*

---

### HOME ADORNMENT.

To make home attractive and worthy to be remembered after we depart from it, to secure for it its best and most lasting influence, to make all who call it theirs—and especially the young—sorry to leave it and glad to return to it, an atmosphere of genuine comfort and taste must brood over its various appointments. The influences with which we surround our children will attend them through life. If, by the objects of ornament and art with which we encircle them, we beget in their minds a taste for the beautiful, that taste never departs. We cannot measure the effects of early influences, surroundings and associations. For good or for evil, they color our whole after life. The son carries from his father's house good or bad tastes, good or bad habits, good or bad temper, and by and by his own home feels the result. A generation later, other young families are organized with their heritage of gentleness—sometimes called gentility—or vulgarity.

We have been reflecting upon the importance of doing all in our power for the adornment of our homes, in view of the influence which is thus exerted. We have been led into this train of thought by several visits which we have lately paid to establishments famed for the beauty of their household goods. To none were we more attracted than to the great furniture house of Mr. L. P. Tucker, (formerly Edw. W. Baxter & Co.) 684 Broadway, corner of Great Jones street. We have surveyed its treasures from basement to roof. We have seated ourselves in a hundred quaint and curious easy chairs, have gazed in wrapt admiration upon the wonderful carving and not less wonderful painting exhibited upon the artistic productions of his factory, and have confidently asserted that a taste for true art can as surely be cultivated by living in the presence of works like these as by seeking the companionship of Reubens or Praxitiles.

If we were to undertake to speak, ever so briefly, of what we saw and coveted, our space would fail. We can only allude to the goods in the most general way. We shall have to content ourselves with the remark that the great warehouse contains furniture enough to supply a good-sized city, and that we saw no article which was not conspicuous for solidity, elegance, and good taste.

In reference to the house itself and its method of doing business we must offer a few words. It is well known as one of the largest manufacturing houses in the United States, if not the largest, and it bears a highly honorable name. It devises and originates new and attractive styles from year to year. It maintains a successful competition with the best American establishments, by the fidelity of its products and its fair and reasonable prices.

We are no stranger to Mr. Tucker's method of conducting his great business, and in that method we discover the groundwork of a success which might otherwise be deemed wonderful. It is not a secret, and yet there are thousands of tradesmen in New York to whom the method is unknown. The rule called "golden" bears supreme sway in the establishment whereof we speak. No piece of furniture is sent out on a mission of false pretence. Nothing is allowed to go forth of which cannot be said, after years of solid service—that article has justified our highest expectations. One thing more. If you buy a piece of furniture here, it is conveyed to your house and noislessly placed in position. The men in attendance then inquire if you would like to have the situation of any of your furniture changed, or if any little repairs are needed about the house. If a castor happens to be off, or a piece of moulding is loose, the injury is promptly repaired without cost. Mr. Tucker has two excellent pamphlets, one a beautifully illustrated catalogue, the other a work entitled "Hints to Housekeepers," either of which will be sent on application.

---

### McCOMBER'S PATENT LASTS AND PATENT BOOTS AND SHOES.

Almost every grown person has a vivid recollection of the discomforts of bunions, corns, soft and hard, to say nothing of the prolonged tortures of ill-fitting shoes and boots, resulting from the use of lasts made after the absurd fashion of a hundred years ago, with a uniform shape, as if every foot were made in the same mold; whereas, scarcely any two feet are alike in shape or size. McComber's Patent Last, recently introduced to the notice of the public, is a perfect resemblance to the human foot, which ought to have been the model for making and shaping lasts, but which has been strangely overlooked heretofore, or entirely disregarded in the desire to make the foot as small as possible, thus flattering the vanity of the wearer at the expense often of lifelong comfort. If the true proportions and outlines of the foot are taken, by studying well its anatomy, a perfect and easy fit is always secured by McComber's method, making corns and distortions impossible, because the fit is comfortable from the start, instead of that long and wearying and torturing "breaking in," of which nearly every reader has had experience, the very thought of which makes one almost shiver. It is not possible for any intelligent person to be willing to have any other shoe after having ordered one made on the principles above alluded to, because, with an hour's wearing, there is such a feeling of ease and support to the whole foot that one almost forgets that the old shoe has been discarded.

As the new last is a perfect resemblance to the shape and set of the foot, it gives a shoe which is natural to the wearer, and nature is always beautiful. But such an accomplishment has been attained at the expense of long years of observation, study and experiment, involving a large outlay of money, and if parents will take advantage of the information given in this article, and have the shoes of all the family from the age of six years made accordingly, the next generation will have reason to remember with gratitude the name of Joel McComber, to whose patient study they will owe the blessing of entire exemption from distorted feet and torturing corns. Nor is this all. One of the greatest health promoters is active, vigorous walking in the open air every day. This cannot be pleasurably done with deformed and aching feet and toes; hence is often omitted when imperatively demanded; and when every step is taken with discomfort, if not actual pain, another result is inevitable—the natural gait is altered, and that youthful spring is lost which gives to bodily motion its litheness and its beauty, to be replaced by that halting and limping and restrained locomotion which is an inseparable attendant on corned and bunioned and distorted feet. In view of these facts, the reader is required to give special attention to the following article:

THE TRUE METHOD OF CONSTRUCTING LASTS, BOOTS, AND SHOES. By Joel McComber, Inventor and Patentee of the "McComber Last;" also, of the "McComber Patent Glove-Fitting Boots and Shoes." Office, 21½ Spruce Street.

Invention has ever been the offspring of necessity in supplying the wants, alleviating the woes, and advancing the highest welfare of the human race. To meet the ever-increasing demands of a more perfect civilization, every age has given abundant opportunities for invention, and doubtless never has there been a time when the world has been so disposed as now to seek and require improved products of industry and skill. As evidence of this fact, witness the improvements in all the departments of active life and business. The constant tendency of the age is to seek for something better, and more rational views of its needs are being cherished and entertained. Hence the requirements for better food and better clothing, more commodious dwellings, greater domestic conveniences of every sort, better products of artisanry, better machinery to do work at less expenditure of motive force. Questions of convenience, economy, and health are becoming more and more matters of serious import, and I freely indulge in the opinion that there is no article of dress which more vitally concerns us than that for *our feet.* Who can not recall the painful experiences coming from wearing improperly-constructed boots and shoes? Pinched and cramped feet, deformed with corns and bunions, are living witnesses of abuse in this particular.

How many have lived since the days of the sandaled prophets who have not been tortured by misshapen and misfitting boots and shoes? Ignorance and tradition have wedged our feet into coverings so at variance with their natural shape and requirements, that we are scarcely better than a nation of cripples.

As to the unseemliness of distorted feet, produced from wearing misfitting boots and shoes, and as to the uncomeliness in the shapes of the latter, there can be no exaggeration. My attention began to be drawn to this subject years ago. From the very fact of my avocation (having been a practical last-maker and shoe-maker for more than a quarter of a century), I have naturally been led to observe, and particularly has my mind been drawn to the study of the anatomical structure and action of the human foot. I have observed much, and some facts derived from these observations are no less remarkable than true. Men's feet, as a general thing, are more distorted than those of the other sex. This is accounted for from the fact that the material of which their boots and shoes are made, being firmer and more unyielding, the feet have been compelled to conform to the unnatural coverings, whereas in the other instance the more pliable material has been made to yield, to a great degree, to the natural shape of the foot.

In the first case, in the unequal contest with shoe-leather, the feet have sadly suffered; while in the second, the feet have suffered less and shoes far more; and not only this, the wearing apparel for the feet is rendered most ungainly in appearance by being wrenched from its original shape in the endeavor to conform to the feet. These facts most forcibly argue the necessity of a modification in the common form of *boots and shoes*, or rather the adoption of an essentially different shape at once more comely and more comformable to the foot, as nature has made it. The fact is, if we would secure the triple advantage of comfort, comeliness, and durability in the wearing apparel for our feet, we *must* have these coverings made in the proper shape as herein subsequently set forth.

It is with no ordinary pleasure I offer to the public a desideratum which has never hitherto been supplied, and I must add that it is with cheerful confidence I anticipate a welcome to this reform which has been inaugurated and already met the approval of an appreciative public to the extent it has been tested. Many have sought in vain the luxury of a good-fitting boot or shoe; they have sought in vain relief from the discomfort of wearing ill-fitting ones. They have resorted to the expedient of having lasts made specially for their use, but without satisfaction. I am sure all such will welcome the introduction of a form and *style of Last* which is constructed upon scientific principles, and made in all respects to meet the requirements of the case. Have parents ever considered how desirable it is to preserve the natural form and beauty in their *children's feet*, by clothing them properly?

How almost invariably are they deformed by improper and distorting pressure! Being subjected to this harmful pressure at an early age, the tender bone and muscle has been compelled to assume an unnatural shape, and in all the future years this cruelty must be atoned for by an unremitting and unrelenting penance.

Who can doubt that *walking* would be far more commonly practiced—I mean for healthful exercise—if it were not attended with so much inconvenience? Many of our soldiers actually broke down in their feet during the recent war, as is well known, and hundreds of thousands were rejected on this very account.

Properly constructed boots and shoes would have prevented this.

Again: a moment's reflection must convince any one that boots and shoes, so made that no undue strain is brought upon one part, while scarcely any or none is exerted upon other parts, will last two or three times as long as those now commonly made. Hence there is a great economy in the expense from having them rightly made.

The following is a brief description of the principles involved in my invention of a new and useful method of constructing Lasts, Boots, and Shoes, for which I have Letters Patent of the United States.

The object of my invention is to produce a boot or shoe which will conform to the anatomical structure of the human foot, so that the boot or shoe, if of proper size, will fit the foot snugly but comfortably when first put on without going through the present disagreeable process of breaking in the shoe to the foot. My improvement is based on a discovery, which is the fruit of a careful investigation of the structure and formation of the human foot.

In order to carry out my invention, I construct a Last with an outside ball and shank projecting laterally more than usual in ordinary lasts; the shank is also lowered to conform more closely to the shape of the foot. The instep is located upon a line drawn from the centre of the heel to the inside of the great toe, as it

Fig. 1.

Fig. 2.

is in the properly-formed human foot, and overhangs the sole of the foot. The inner ball of the Last also overhangs the sole. The inside shank or hollow of the

Last is likewise made to conform to the shape of the foot.

My invention will be better understood by reference to the accompanying drawings, in which Fig. 1 represents in full lines a diagram or plan of the natural human foot; that is, one undeformed by wearing improper boots or shoes. The dotted lines in this figure show the outlines of the sole of my improved Last.

Fig. 2, a view of the underside of a Last constructed according to my improvement; Fig. 3, a view of the inner side of my improved Last; Fig. 4, a view of the outerside of the same; Fig. 5, measures of the foot for Lasts.

Fig. 3.

Fig. 4.

In the natural human foot a line drawn from the inner side of the heel, and touching the ball of the great toe, will also touch that toe throughout its entire length.

In Fig. 1 I have, however, shown the great toe as very slightly drawn in, as it naturally would be, when a shoe was worn. This line A above mentioned I call, for the sake of distinction, the base line.

A line B, drawn from the outer side of the heel parellel with this base line would pass through the third toe B, usually near its centre. This line, for the sake of distinction, I call the sole line.

A line C, drawn from the centre C of the heel to the inner point A of the great toe, would pass through the instep D of the foot. This line I call the instep line In the natural human foot nearly, if not quite, one-third of the foot lies outside of the sole line B, and the tendency of the pressure and spread of the foot is all outward. This will be seen by observing the bare foot in walking.

The great defect in the ordinary shoe is that it is formed on some arbitrary notion of symmetry irrespective of the natural formation of the foot, which is distorted into the shape of the boot or shoe. This arbitrary form generally combines an instep near the centre of the shoe, an outer line of the sole nearly straight, and a toe pointed or inclined inward from each side.

The consequence of this mode of construction is that the great toe is deflected inward, and the foot thus deprived of its support, while the outer side of the foot is drawn inward also. This narrowing of the base of support renders the walk unsteady, insecure, and produces corns, bunions, and contracted toes. The common shoes after being worn will have become tread over outside by the effort of the foot to widen its base of support.

I construct the sole, E, of my improved Last to conform closely to the shape of the foot, as shown by the dotted line in Fig. 1, making it, of course, slightly longer to leave room for the play of the toes in walking, as is usual.

The sole, it will be observed, is nearly flat across the ball, but is curved in other parts to conform to the foot, as shown in the drawing.

I form the instep D full or bulging, so as to overhang the inner side of the sole or the base line A, Fig. 2. The inside ball F is enlarged, and overhangs the inner side of the sole until it reaches the first joint of the great toe (see Fig. 2), and is thicker all the way to the end of the great toe than the corresponding portions of the outer side of the Last, which are thinned down to correspond with the diminished thickness of the foot on that side.

The outer side of the instep and waist is hollowed out so as closely to conform to the shape of the foot.

The outer shank G and ball H are lowered nearly to the same plane as the outer line of the ball and heel, and extended laterally as far as the foot does, thus giving a bearing for the foot on the sole and shank of the boot or shoe, as it should have. The boot or shoe being a counterpart of the Last, needs no description here.

The workman should, however, be careful to work to the outlines of the Last, wet the upper when lasting, and last the boot or shoe well, taking all the stretch out of the leather.

I thus produce, from nature's own model, a boot or shoe that presents a symmetrical and beautiful appearance from the start without breaking in the shoe to the foot, or straining or injuring it, and one that will retain its proper shape until worn out.

I have often wondered why it was that so little has been done to secure clothing for the feet, so as to combine the advantages of a "good fit," a graceful and symmetrical boot or shoe, and, above all, an apparel that would permit the healthy growth, enjoyment, and proper action of the feet.

From the common "corn and bunion doctor" to the educated "Orthopædist," we hear next to nothing of "Pedal Hygiene," or the art of preserving the feet, which ought to rank as at least a semi-science. We are treated to the most liberal promises to cure (often not fulfilled), but hear nothing and know nothing of prevention. With a few unimportant exceptions, and for reasons not apparent, medical men have purposely, or unintentionally, neglected this productive little corner in the field of Hygiene. Possibly they thought it *infra. dig.* to dabble in the craft of the common cordwainer, or mayhap could not humble themselves to bow at the shrine of St. Crispin! But the true physician, who also understands his duty, can not afford to slight any branch of knowledge, seience, or art, which can be made subservient to the relief of suffering man and beast.

Turn we to the craft itself, and ask what it has done for humanity in the direction indicated? Not in its whole history anything, except one or two attempts somewhat creditable, doubtless, to the authors, as theorists, but ineffectual when brought to the test of practical utility; and thus we see the feet have been less or more deformed in all ages, among civilized nations, in nineteen out of twenty persons. But deformity is a mitigated evil, compared with grave and often fatal local and constitutional diseases arising from a habitual disregard of the laws of health, as affecting the feet. Among some of these may be mentioned corns, bunions, ingrowing nails, riding and contracted toes, sprained and weakened ankles, club feet, headaches, etc , etc.

Life becomes miserable, the temper odd and cynical, and man who prides himself in his erect position, has often been tempted to congratulate the horse on which he rides on his superior mode of progressing, the blacksmith being several thousand years in advance of the bootmaker in their respective crafts!

To make a good and true fitting boot and shoe, the lasts commonly in use must be discarded, as they have invariably been manufactured in defiance of the anatomical structure of the foot. The inveterate fault, never scientifically and radically cured, is still the practice of constructing the boots and shoes as if the outside of the foot was as firm and unyielding as the inside arch! This is by no means the case, as common observation will show, and is proved by any half-worn boot or shoe picked up at random. The human foot presents two arches—one transverse—resting by one end on the ball of the great toe, and by the other end on a point corresponding with the junction of the little toe and the bone with which it makes a joint; when the foot is planted firmly and supports the half weight of the body, it will be seen that the outer butment of this arch sinks. Again, the foot makes an arch longitudinally, the outside, or outer edge of the foot sinking and inside remaining firm. The common last makes no systematic allowance for this, and consequently the boots and shoes "go over," forming distressing corns on the little toe and side of foot near its joint, while the toes are crowded in a mass. In the meantime the great toe is drawn in, causing a bunion, occasionally the quintessence of misery; and bipedal locomotion thus becomes difficult, ridiculous, or impossible. From the earliest records of history we hear of whole armies baulked or vanquished by faulty shoes, supplied by a heedless or ignorant commissariat. The modern proverb, or toast, was well known to the ancient Greeks:

"Short shoes and long corns
To our country's enemies."

The stubborn fact must be admitted that ninety children out of one hundred are born less or more predisposed to "talipes" or club foot. This tendency science must correct in the race, otherwise it must be aggravated by inheritance. It is easy to see that in the majority of cases the *flexor* muscles of the calf of the leg are more than a match for the extensors. If this discrepancy is not counterbalanced it leads to a grave error in boot and shoe making. It will be found that a line let fall through the centre of gravity, on each side of the body, which ought to impinge on the boot sole at a point which would exactly bear one-half the superincumbent weight, has, from time immemorial, been made to strike a point *outside* of the true point. By a careful examination of this matter, I have estimated the error at from half to three-fourths of an inch! How many men and women have broken their hip joints and shoulders, not including minor casualties, passing along over pitching sidewalks and beveled corners, on account of this seemingly trifling blunder, statistics will never tell! As at present commonly made, boots and shoes are well designed for our pitching sidewalks and beveled corners to break the necks and crack the skulls of old and young by shooting them into the gutter.

But every evil cries aloud for its remedy—sooner or later to be supplied; and every demand has a tendency to be associated with its adequate supply, and such is the case at last in the matter of footgear.

Any person of common understanding who will give this subject a careful investigation, will at once come to the conclusion that this Last amply supplies the above mechanical and physiological requirements, and revolutionizes the boot and shoemaker's art, which also becomes a science, and a humanitarian one at that.

After years of study, perseverance, and experiments, I have perfected a Last which will, aided by thorough workmen, unerringly yield a boot or shoe emphatically to "fit the foot," and even correct deformities, when susceptible of correction or cure, short of the aid of surgery—have, indeed, laid down a deeper and surer foundation for society, and improved the understanding between man and his mother earth, between whom there has so long been so serious a misunderstanding.

The principle combines equality of pressure, and therefore "a close fit," with beauty and symmetry. The tendencies of gravity and the play of the muscles are scientifically respected. In fact, when a man has on a pair of the McComber boots, he is himself again, and stands perpendicular with the world and square with everything and everybody.

I invite the attention of Boot and Shoe

Manufacturers of all classes of goods, from the finest ladies' and gentlemen's boots and shoes to the farmers' and miners' heavy kip and stoga—not forgetting to invite the attention of the medical profession in an especial manner to its importance from the hygienic stand-point.

This is no mere fashion of a season, but an everlasting principle applied to remedy an error in the construction of boots and shoes, which has caused more suffering than any other error that can be named.

Boots and shoes for men, women, and children, of all classes of material, made in factories and sold by the case, can be used by retail dealers to secure perfect fits in every instance, as a glove fits the hand, by having the different sizes and widths on hand.

REASONS WHY.

Why are the patent boots and shoes made on the McComber principle superior to all others? For at least nine reasons:

1. They are made to fit the feet, whereas according to the old plan, the feet were *made* to fit the boots and shoes, an absurd, monstrous, and pernicious custom, which has made partial or complete cripples of one-half of the human family.

2. Because the anatomy of the lower limbs, with their muscles, joints, and ligaments, as well as the feet, being carefully studied by Mr. McComber, he has succeeded in supplying foot gear that enables mankind to walk forth with that ease, grace, and confidence and dignity of carriage with which the human form divine has been so richly endowed by nature, and so ruthlessly robbed by the blundering vagaries of unscientific and arbitrary men.

3. Because the boots and shoes are made to fit easily from the first, the *Last* being better able to bear the brunt of breaking in than the feet themselves, tender organs at best, though the latter practice has been in vogue until now—a practice the cruelty of which millions will attest, and which often revives the recollection of the veritable ancient boot of the days of persecution.

4. Because they are twice as durable, the pressure being equal at all points, whereas in the old boot or shoe, friction and inequality of pressure caused corns and premature wear and tear at particular points, and also endangered the feet from freezing in winter.

5. By following nature the model is more graceful, elegant, and symmetrical; though the old school of shoemakers thought that the venerable dame was no match for them, but a trial of McComber's boots will show that for the first time the centres of gravity in boot and shoe making are settled by him so precisely, that the wearer can now keep his balance, standing or walking.

6. Because easy-fitting boots and shoes are indispensable to health and good looks, and, it may be added, to the moral deportment of mankind. It is possible "The Army in Flanders" would have kept the commandment, "Thou shalt not swear," better, if Joel McComber had supplied them with boots and shoes, as corns, bunions, etc., are unknown to the wearer of them.

7. Because the McComber boot tends to prevent as well as cure the manifest inclination to club feet, a terrible distortion, which is on the increase, and which the best physiologists declare possible to be entailed by hereditary transmission even when artificially originated by run-over boots and shoes.

8. Because the wearer can walk faster, farther, easier, better, more comfortably, more gracefully than in any other boot or shoe.

9. Because by wearing the McComber boots and shoes, the inveterate abuse of the human feet is effectually prevented, and the image of God, thus far, preserved from deformity, as there is no cure so perfect as the absence of the disease. It is, in our judgment, positively wicked to destroy the symmetry and graceful appearance of the natural foot, when by clothing it intelligently its beauty as well as its usefulness may be preserved as long as those of any portion of the human frame.—[*Copyright secured.*]

### AMERICA LEADS.

In our search for useful information we called at the store of the Meriden Britannia Company, 550 Broadway. Here we saw a lot of goods which were about to be shipped to France. This looked strange to us, for we have been accustomed to believe that French artisans must, of course, excel our own in matters of taste. Further inquiry satisfied us that, for graceful designs in silver and plated ware, America not only leads the world but practically supplies it. Our products in this line are at once more excellent in quality and durability, and more conspicuous for graceful symmetry. These facts secure for the American manufacturers the trade of the best foreign dealers, as well as frequent orders from foreign travelers for goods for private use.

We sought and obtained many interesting facts during our half hour in the great establishment alluded to, having made the acquaintance of Mr. Wilcox, one of the officers of the company. From him we learned that the "Meriden Britannia Company" was organized about thirty years ago, for the manufacture of a kind of table ware, which has since gone into disuse—Britannia. At that date little was known of electro-plating in this country. That process has revolutionized the trade. It has substituted for the old goods the elegant modern productions, made of hard metal and coated with pure silver. The Company has, however, adhered to a name made honorable by a long period of business success. That success may be approximately estimated when we state that from a small beginning it has grown until its annual sales reach $3,000,000, with seven factories, the largest, at West Meriden, Conn., being about seven hundred feet long. To-day it manufactures more pieces of plated ware and uses more nickel-silver annually than all other manufacturers in the United States. Its ingenious workmen have devised a method by which those portions of certain articles of table ware most exposed to attrition shall receive a much greater thickness of silver than the parts less liable to wear. Thus the points of forks and spoons and those portions which naturally come in contact with substances calculated to denude them of their precious covering, are protected by a silver film three times as thick as other parts receive. That this method of plating is practicable and successful is attested by the Judges of the American Institute for 1873, who carefully examined the goods and declared that they were probably the best of their kind made in the world.

Another important specialty is the manufacture of porcelain-lined Ice-pitchers. The exterior of these pitchers compares favorably with the most graceful designs in silver. Within the water receptacle is a solid, hard metal structure, coated with a pure white enamel. As the enamel is made from silex, we have, in effect, a pitcher of flint! By the ordinary method, the interior wall of the ice-pitcher is made of tin, copper and antimony, and the bottom is soldered in with a solder of lead and tin. Galvanic action is certain to be established, and the water can not escape impregnation.

Our readers will do well to examine the goods at 550 Broadway, although they can scarcely enter a well-appointed house in which they may not be found. The Windsor Hotel was supplied here, as were the City of Pekin and the steamers of the Pacific Mail line. But at the establishment of the Company they can obtain elegant ware sufficient to garnish a hundred cities and supply the tables of all the ships that float.

---

### THE RIGHT COURSE PAYS.

A new exemplification of this truth is manifested to us almost every day of our lives. During our long career we have known hundreds of young men who have risen to positions of power and influence because of their carefulness in little things. We have watched the downward course of other young men, with keen regret, and have seen splendid talents run to waste and years of education thrown away, on account of some tendency to unfair dealing. No one knows better than the successful merchant how largely it pays to be honest. To secure a reputation in this direction, will almost surely lead the business man to a competency. A good name is said to be better than great riches, and it is clear that, in mercantile affairs, it is almost certain to lead to them.

We are thinking of Richard Meares, of Sixth Avenue and 19th street, as we write these lines. He is quite a young man, and yet he has long been a prominent and successful merchant. The space occupied by his business is equal to that required by five ordinary stores. The stock of goods is enormous. On the first floor are the salesrooms, in which are displayed almost everything which can be thought of, while above and below are stored the goods from which the stock is hourly replenished.

We have been looking the place over to determine whether humanity could be benefitted by our observation. We think we can point some useful morals by calling attention to it. We know the belief is very wide-spread that red-flannel undergarments are healthier than those of any other color; that perforated buckskin shirts, worn next the skin, protect the vital organs more perfectly than any other covering; that "chest-protectors"—a comfortable pad, covering the lungs—protect the breathing organs from sudden chills and congestions. These goods are kept in great abundance by Mr. Meares.

Beside these, there is an endless variety of useful things which no man could well number. There are rooms devoted solely to the cutting and making of dresses and cloaks for ladies, in which, when the customer is in haste, an elegant suit can be made up in a few hours. This is especially true of mourning goods, in which specialty the house does a very large business. The best *modistes* are employed, and the products of this department seem to be characterized by genuine artistic grace. Another room is all ablaze with wonderful hats for ladies. Feathers, and ribbons, and flowers and birds, gladden the eye in all directions. In an inner apartment, concealed from public gaze, were some exquisite fabrications in bonnets, dresses and cloaks, from France, selected by the foreign buyer of the house, and which can only see the light on the day of the autumn "opening," soon to occur.

In the line of warm woolen and merino goods for the approaching cold season, the stock seems well nigh endless. There are splendid all-wool undershirts and drawers not only for men's use, but also for ladies and children. How much cold and suffering and disease will be warded off, we reflected, by these warm and fleecy goods! And then the gloves of every style and price, from the dainty little affair, made from soft kid and worn by the cunning fingers of the child, through all grades and sizes up to that wonderful structure, with its long row of fifteen or twenty buttons, designed for the full dress party glove of the finest lady in the land. Then the hosiery, with more stripes than the flag and more colors than the rainbow, endless in variety and most comfortable in texture.

But we cannot undertake to enumerate. The great stores contain nearly everything which ladies wear and much which gentlemen could not enjoy life without. The place is full of busy buyers and active sellers. To wait on the first named class, one hundred and twenty-five of the last mentioned are employed. This speaks well for a trade which commenced with four clerks thirteen years ago and occupied one moderate sized store. What is the secret of this growth? Simply that the goods are honest and genuine, the prices reasonable, and no customer is taken advantage of. The right course is pursued, and the right course pays.

### ADULTERATIONS IN FOOD.

THERE is no question but that the quality of the blood in our veins and the condition of every tissue of the body is greatly influenced by the character of our food. It has long been an accepted theory that the nervous system and the mental powers may be refined, elevated and strengthened, or demoralized, lowered and weakened, by the food consumed. We can poison the fountains of life just as surely through the stomach, by consuming unwholesome food, as through that more direct channel, the lungs, by breathing a poisoned atmosphere. The expert physiologist can usually determine the character of the food taken and the air inhaled, by the condition of the muscular system and the tone of the complexion. The poorer classes in our large cities, who are compelled to buy their food in small quantities, and to make a little go a great way—to whom bulk appears to be more essential than excellence—exhibit, to the practiced eye, in their physical condition, the effects of what is called cheap food. They are flabby and pale and unwholesome looking. Their vital powers are feeble, and thus they offer only slight opposition to epidemic and other diseases. If a few simple injunctions could be universally obeyed, the world would be astounded to see how much suffering could be averted, how greatly human life would be prolonged. A temperate and systematic life, abundant sleep, pure food, pure air, bathing, exercise—this list embraces pretty much all the essentials, and every item *is* an essential.

But this brief chapter is devoted to only one of the half dozen topics enumerated. The substances which we take into our stomachs to nourish our bodies must be pure, wholesome and nutritious, or we suffer in consequence. We may be deceived, but the knowledge of the stomach is instinctive and as unerring as chemistry.

There is no middle ground in the selection of food ; we must have the best, if we would be well. We cannot drink colored tea and have sound nerves. We cannot swallow plaster paris in our powdered sugar, and enjoy good digestion ; we cannot eat bread made from grain which has soured—which has lost much of its nutriment by semi-decomposition and has been "doctored" into a semblance of sweetness—and have good blood. All other conditions may be fulfilled, and yet, if our food is unsound our bodies will be unsound.

We had a long talk on this topic, the other day, with Mr. Park, the head of the house of Park & Tilford. Few of our adult readers can be unfamiliar with the names of these well-known dealers in articles of food. For thirty-five years they have furnished the best tables in New York with the products of every land. Honestly and earnestly they have exerted themselves during all this period to supply their numerous customers with the purest and best of everything. If by any means a bad article creeps into their stock its doom is sealed, and that doom is nothing less than destruction. A chest of tea, costing a hundred dollars, is as ruthlessly consigned to the great furnace, if damaged or "doctored," as would be a box of cigars which had been dishonestly made. It is costly fuel, but, in the end, it would prove more costly food. A cask of old port wine, valued at $1,000, would find its home in the North River, if made of drugs or rendered fiery by poisons, as speedily as would a bottle of vinegar made from vitriol. By this system, and the financial ability to carry it out, the house has secured a reputation which will live long after the present members of the firm have passed away. Mr. Park communicated to us many important facts which we would gladly repeat to our readers. He explained the methods practiced and the care exercised by them to secure the purest and best of everything. He told us with what scrupulous fidelity the wines which they receive from foreign vineyards are prepared from ripe and selected fruit, and how absolute is their knowledge of the purity of this class of goods before they consent to offer them for sale. Alluding to what is known as port wine, he remarked that there had never been an hour since the establishment of the house when they had not been supplied with the pure fermented juice of the grape of Portugal, rich in color, ripe in age, and absolutely free from any element or admixture not obtained from the crushed grape. Of course the price varies according to the amount of care used in the selection of the fruit and the number of years during which the wine is stored before using. But with them, these were the sole considerations regulating the price. All were pure; all were the simple and unperverted product of grapes.

The vast growth of the business of this great house is at once the record and the exemplification of its honorable course. It employs one hundred and twenty-five men in attending upon its customers. It keeps thirty-five horses actively employed in delivering its goods. It occupies a large store on the corner of 21st street and Broadway, and another on the corner of 38th street and Sixth Avenue. Besides these, there is the old establishment on the corner of 9th street and Sixth Avenue, and the great edifice now in process of erection near by, which will be numbered 118, 120 and 122 Sixth Avenue, and will be complete and perfect in its way. As workers with us, and in a very important direction—the supplying of pure foods and drinks—we cordially and conscientiously recommend this house to all who would preserve their bodies from premature decay, by supplying the waste which is ever going on in the human system with the best and purest of everything.

---

### HEATING AND VENTILATION.

In an article in this number we incidentally give a list of essentials, without which health cannot be secured and human life prolonged. One of these essentials is pure air. Many physiologists will declare that it is the most important of all. As portions of the blood are renewed or impaired by each expansion of the lungs, it is evident that the excellence or the poverty of that fluid upon which life and health depend, must be largely governed and regulated by the quality of the atmosphere habitually inhaled. It is not difficult to secure good air during the season of temperate weather. The trouble comes as the cold sets in. If our bodies were in constant motion during our waking hours, we could live and be warm through our winters without artificial heat. But by far the largest part of our people are compelled to pass much of their time in-doors. To all but the active out-door laborer heat other than that supplied by the combustion of food is necessary. Many whose life by day is in the open air are forced, from the circumstances surrounding them, to breathe air artificially heated at night. How then shall this artificial heat be supplied?

In small dwellings where few apartments have to be warmed, and where cheapness is desired, fire-places, stoves and heaters can be employed to advantage. In public buildings and first-class houses, the choice is between the use of steam in some form and hot-air furnaces. The system known as "direct radiation," in which great clusters of pipes or plates receive the steam and radiate it to the apartment in which they are placed, is much in use. But we deny its healthfulness. The air of an apartment thus warmed soon becomes oppressive and almost insupportable. We go out from a room thus heated with a sense of tightness across the forehead, which suggests congestion. We feel that we are absolutely breathing over and over again the same old, lifeless air. Moreover, if we sit in air which has had its vitality exhausted, it must be at a higher temperature than if full of vitality.

The hot-air furnace, as ordinarily constructed, is the most dangerous heating appliance known to civilized man. An undertaker in a large city was asked at what season of the year his business was best. He replied that he was kept about as busy at one time as another, though the winter paid best. In the summer the poor folks died off, and in the winter the hot-air furnaces killed off the rich ones.

The best heating device which we have ever examined is that called the "Union Steam and Water Heating Company's" system, which is employed in the Congressional Library at Washington. We have sat in the great halls of that library for hours at a time, in cold and stormy weather, and have got up from our work with a sense of freshness and elasticity which can only attend labor in a pure atmosphere of agreeable temperature. We have conversed with Mr. Thomas Angell, of Angell, Atwater & Co., 706 Broadway, New York, and have familiarized ourselves with the workings of the system. We have frankly assured the manufacturers, as we now assure our readers, that we should, with our present sources of knowledge, invite these gentlemen to supply our house with their system of heating, if we were about to build. It is as near perfection as the art has produced, so far as we know. It secures to all parts of the house pure air drawn from without, and warmed, without being burned, by coming in contact with enclosed radiating steam-chambers having a multitude of projections upon them by which the heating process is greatly facilitated. The apparatus is practically self-regulating, acting at all times automatically to lessen or increase the heat, regardless of the intelligence of the attendant. The edifice warmed by it is sure to be a wholesome one to live in, if an appropriate place of exit is afforded for the escape of the air after it has been used.

We have not the space to devote to a more complete description of this valuable device. We may find occasion, however, to do so hereafter, as well as to allude to a remarkable Kerosene stove for cooking and heating, about to be given to the public by Messrs. Angell, Atwater & Co. But we earnestly advise all who seek the best heating device to consult this well-known and highly honorable house.

---

### AN ENCOURAGING OUTLOOK.

A WELL-KNOWN gentleman of this city, manager of the leading clothing house here, remarked to us a week since that his establishment was selling all classes of clothing cheaper now than at any time since 1861. An examination of the goods and prices not only convinced us of this, but satisfied us that while the prices had materially fallen, the standard of excellence for which the house had long been celebrated, had been honestly kept up. The concern of which we speak—that of Messrs. Devlin & Co—has been known to New Yorkers since 1843. Its constantly increasing trade has enabled it to extend its facilities from year to year, and it has long been recognized as the leading house in its line. Its business was commenced thirty-one years ago in the building at present occupied by the *Evening Post*, a region long since abandoned by most branches of retail trade. It now demands two large stores—one on the corner of Broadway and Grand street, the other on the corner of Broadway and Warren street. That honorable and honored citizen, so well known and so highly respected during his long business career among us, Mr. Daniel Devlin, was its founder. At his decease, in 1867, the immense business was continued by the surviving partners, the style of the firm remaining unchanged. As it was originally, it is now, and will doubtless long continue—"Devlin & Co." The head of the house is Mr. Jeremiah Devlin; his sole partner is Mr. Robert C. Ogden.

It was no plan of ours, when we began this brief article, to say complimentary things concerning a house whose reputation is firmly established, and which can neither be increased nor diminished by the attitude assumed by the public press. But one fact comes up in our mind which may be very properly introduced here.

We have a relative residing in a far-off Southern city. Twenty years ago, on his way thither, he purchased an outfit at the store of the Messrs. Devlin. The articles fitted him accurately, and were pronounced models of grace and ease. He carefully preserved for future use the tags or tickets which were attached to the several garments. A year later his wardrobe required some replenishing. He mailed to the house a copy of the appropriate tags and indicated the style of cloth which he preferred. The articles came promptly. Thus he continued from year to year, until the war broke out and intercommunication was precluded.

For five years, our friend assures us, he would have been as shabby as any other man within the Confederate lines had it not been for the superior wearing powers of his old clothes. The accumulations of several years stood him in good stead. While his neighbors were paying $5,000 in Confederate currency for a decent coat, he drew on his cast-off reserve and made as good an appearance as the best of them. As soon as postal communication was re-established with the North, up came copies of that original tag. He has kept up the system ever since, and assures us that he has never had a misfit, never an unsatisfactory garment.

The house has a system of self-measurement, at once simple and accurate. If your home is remote from New York, you have but to address them a postal card asking for their pamphlet, and you will receive it. It tells you how to take your own measure, or that of your little boys. It gives you pictur es of graceful garments, and, when the book is mailed, samples of cloth will accompany it if you ask them. Then the goods will be sent by express and the bill collected. The price will be reasonable, covering only a moderate margin of profit. Our word for it, you will be abundantly satisfied with the transaction.

We have wandered somewhat from our purpose. We intended to give some description of the two great establishments, and to speak more in detail of certain articles especially adapted for protection during the approaching season of frost and snow. We had designed to allude particularly to the comfortable Ulster Overcoat, as modified and rendered elegant by this house. But our limits forbid. Perhaps we may find space next month. Meantime, let our readers ponder what we have here said, and act upon the hints which we have thrown out.

---

### GLENCOVE.

We passed the weeks of August at the delightful spot bearing this name, having our home at the residence of Mr. T. T. Jackson, near the water's edge. A member of our family who had not been quite well for several months gained precisely one-third of a pound of flesh per day during our stay. She lived in the open air, exercised very freely by taking long walks, ate heartily, because the appetite was stimulated by the bracing air and the constant motion, and, also, because the food was of the purest and best, such as milk in abundance, butter made daily, vegetables gathered the moment before they were cooked, and "cottage cheese," that most nourishing and flesh-producing of food's. The free use of the water of an iron-spring on the premises aided digestion, and rapidly built up the waste. We do not know of a more delightful summer resting place than Mr. Jackson's.

---

### A VALUABLE DEVICE.

For some months past we have, at the request of the proprietor, Mr. E. H. Gouge, published in our columns a description of "Outwater's Vaporizer." Within the past few weeks we have had occasion to try some experiments with this instrument, and to test its merits in an important manner; we now propose, therefore, to give our opinion thereon to the public.

The Vaporizer is a small metallic boiler, with its stand. The cap of the boiler terminates in a small steeple, which is perforated at the tip on four sides. On this steeple rests, loosely, a hollow arm. By nearly filling the boiler with water, and placing an alcohol lamp beneath it, steam is presently evolved. The only point of escape is at the steeple-tip. Here it passes into the hollow arm, which at once begins to revolve and to discharge steam at the same moment. The steam is projected by centrifugal force to a great distance in every direction, and the atmosphere of the apartment in which the instrument is placed is quickly affected by it.

The uses to which the Vaporizer is applied are, medicating, disinfecting and perfuming the atmosphere in localities where it is desirable to effect these results. That it will very rapidly change the condition of the air surrounding it is evident. The water in the boiler is used as the vehicle for the conveyance, in the form of vapor, of the substances with which the air is to be impregnated. The contents of the boiler are necessarily in a state of complete ebullition before any vapor is evolved. Of course the medicine or perfume which has been added is divided infinitessimally before it is projected from the instrument. It is thrown off with great force in combination with the steam, and its existence is perceptible only to the sense of smell. The air is penetrated with the medicated vapor with wonderful celerity. If you add a couple of drops of carbolic acid to the water in the boiler, and put it in operation in the largest hall or church in the city, you will detect the carbolic odor in the remotest corner of the enclosure in two minutes after the vapor begins to escape. If you wish to impart any particular perfume to your parlor, you may do so almost instantly by adding a minute quantity of that perfume to the boiler and placing the instrument in motion on your centre table. If you would perfume the whole house, you will simply open the doors of all the rooms and place the boiler in the lower hall; you will find that the amount of perfume which you ordinarily pour upon your handkerchief will, if diffused in atoms by this instrument, and borne upon vaporous wings, impart its odor to your entire house, and every person and thing contained in it. As an inexpensive luxury, then, we think it deserves to be spoken of in high terms.

But our interest in the invention is in its uses as a means of disinfecting and medicating. We know that great good may be done by the inhalation of medicated vapors in certain cases. The theory of their application is very simple, and will be explained hereafter. We desire to speak of a practical test.

A member of our family had a sore throat, attended with great difficulty in swallowing. We added to the water in the boiler five drops of oil of tar and a fragment of saltpetre about the size of a hazel-nut. We set the instrument in motion, closed the room, with the exception of a reasonable ventilating space—secured by lowering a window from the top—and compelled the patient to recline comfortably on a lounge, and breathe the air thus medicated for two hours. The sore throat vanished and has not returned.

We have made other tests which we cannot stop to recount. It works magically in distributing disinfectants. A penny's worth of chloride of lime or chloral-hydrate thus employed will disinfect and deodorise more space, we think, than would a dollar's worth used in the ordinary way. The steam—itself a disinfectant—carries it everywhere.

We believe the instrument to be very valuable, both as a luxury and as a sanitary agent, and that it ought to be in every house. In the sick chamber it is wanton to dispense with it, while in the treatment of catarrh, bronchitis, and all diseases of the air-passages, its employment must be attended with very beneficial results.

---

### "THE LEADING NEWSPAPER."

What a wonderful paper the *New York Tribune* is. We take it up regularly six days in a week, and rarely without new emotions of wonder. Here is a large sheet—and not unfrequently two of them—filled with the ablest matter on live topics. Many of its editorial articles would do credit to any magazine in the world. Its dramatic criticisms are the ablest and the fairest which fall under our observation, and in the treatment of special topics it is ever trustworthy and vigorous.

We write these words with the current number—that of Monday, September 21—lying before us. Here are twelve pages of news and literary matter, including the most complete reports of the sermons of yesterday. We turn to the report of the sermon to which we listened—that of the Rev. Robert Colyer—and find every prominent thought uttered by that eloquent divine, here reproduced. Had the sermon been printed from the author's manuscript it would have been less valuable, for it could not have contained the running commentary which sprang to the speaker's lips as new thoughts pressed for utterance. Truly, the mantle of the great founder of the *Tribune* is worthily worn. It is the most brilliant daily journal in the world to-day.

---

### A SUBURBAN CITY.

We do not know when we have met a more enthusiastic gentleman than Mr. W. Jennings Demorest. There is an activity and energy about him which is contagious, and we instinctively think quicker and talk faster in his presence.

He has poured into our willing ears his plan for building up a delightful city at a point which he has selected eighteen miles from Boston. He calls his new city "Vienna." It is on the Boston and Providence railroad, and occupies a high plateau, two miles long and one mile wide, divided in the centre by the iron track. It is between the beautiful villages of Sharon and Foxboro'. This section has certain important advantages for consumptives and health-seekers. Dr. H. J. Bowditch, in a work on Local Causes of Consumption, published in 1862, alludes to Sharon, Mass., as especially likely to be free from lung diseases. The record shows that one-fourth of all who have died in that town for a number of years past were over eighty years of age, and that one-half the deaths were of persons over eighty-seven years.

The causes for this remarkable longevity are numerous. The climate is salubrious, and the elevation gives it immunity from chills and fever and all epidemics. The air of Newport and Narragansett Bay comes to it softened and lacking the sea-side harshness. To our mind another cause of the healthfulness of the tract selected for the new city is found in the fact that a stratum of iron ore underlies it. This metal performs a double office, both of which may be considered of great importance. Recent discoveries in chemistry attest that the best water-filter in the world is made from iron ore, when artificially treated by being heated sufficiently to drive off whatever sulphur it contains. The ore is found to remove all impurities in the nature of albuminoid and nitrogenized substances, as also all lead contaminations, and to present the water absolutely pure, excepting only a small trace of iron. Iron water agrees with everybody, gently stimulating a natural and healthy appetite, and ordinarily affording no unpleasant action.

A few hundred yards east of the new city is a beautiful lake called Massapoag, seven miles in circumference. Here has been erected a fine hotel, accommodating 250 guests. People go there too sick to stand, and speedily take on strength and flesh and buoyant spirits.

The city is being laid out in Boulevards, Avenues and Streets, and is divided into building lots for suburban residences. The widest Boulevard is 150 feet wide, and is projected to have two roadways, with a walk and two rows of trees in the centre. Other parallel avenues, one hundred feet wide, with intersecting streets sixty feet wide, and the whole divided in lots of 50 feet front by 100 deep, together with provisions to be made for a grand hotel surrounded with a park, circular drive, and places designated for school houses and churches, are to be embraced in the plan. Deeds for the lots sold contain stringent covenants against the erection of nuisances of any kind, and prohibition against selling spirituous liquors, etc., thus furnishing a model for a town of which the country may well be proud.

A cultivated and superior class of citizens are destined to congregate here. Artists and literary personages and professional people; the cultivated and the gifted; those of æsthetic tastes and pure lives are the class sought for, and these will form the basis of the new city.

---

### BORN, NOT MADE.

*Poeta nascitur, non fit,* is a phrase first used many, many years ago. It simply means that poets are such by nature, and not by virtue of education. It is unquestionably true of genuine poets as it is of all real possessors of great gifts. For there are some things which you cannot confer upon the man, educate him as wisely as you may. You cannot make him a great musician or a great sculptor, a great orator or a great inventor, if that power which we call nature, has not been before you with great endowments.

We think, at this moment, of an illustration in point. Many years ago a young man of singular skill as a designer, associated himself with a friend and established a house in this city for lithographing and printing. The firm was known as Sarony & Majors. Wonderful pictures emanated from their establishment. Menageries and circuses circulated their marvelous productions all over the land. There was an air of grace and a dexterity of execution about them which startled the beholder. This was the work of Sarony, now the photographer, and the most artistic of artists.

Those of our readers who have never had the good fortune to secure a likeness by Sarony will at once depict in imagination the usual method which they have seen employed in "having a picture taken." They will see themselves screwed into some impossible position, having a big-barreled box aimed at them, and then blinking at a knot-hole for a few minutes which seem like ages. Such is not the treatment received at the hands of this leader of art. You sit or stand or recline, in light or in shadow, with this, or that, or the other view presented to the camera, just as the quick and unerring eye of the artist decides is best. You want a likeness full of character and expression, and having in it something of yourself. But it must be yourself in your best moments, or you will not be satisfied. It must be life-like, but it must be like life in its happiest and loftiest mood.

Just at this point comes in the true artist-genius. Sarony watches you, and discovers, perhaps, that your good looks depend less upon facial regularity than upon the lively, earnest play of feature which is yours in your moments of supremest interest. So he plans to transfix upon his plate that one, fleeting expression; and he rarely, perhaps never, fails. The work which he presents you will be excellent as a likeness, exquisite as a work of art. You will be proud to present it as a keepsake to your friend, and your friend will be proud to preserve it.

It is most interesting to visit Sarony's rooms, opposite the Grand Central Hotel. There you can view some wonderful portraits of celebrities, which do not seem to be photographs, and which are yet too accurate as likenesses to have been drawn by any pencil less unerring than the sun. You are told that they depend for their outlines upon the camera, a mere faint tracing having been secured by photographic process, and then filled in with charcoal. The effect is marvelous. You do not wonder at the reputation which the great artist has acquired ; you admire his achievements, and look upon his fame as a thing of course.

---

### THE JOURNAL CLUBS.

THE business which we so long ago inaugurated of clubbing with other valuable publications has grown to be very large, and seems to meet a real public necessity. We receive the money and become responsible for the prompt mailing of the publication ordered, as well as for our own. The price is lessened materially by the fact that the proprietors of both publications make a very considerable discount. Two journals, having different aims, are thus secured for a small percentage over the price of one.

We have just arranged to send HALL'S JOURNAL OF HEALTH in connection with Demorest's very valuable publications. These consist of DEMOREST'S ILLUSTRATED MONTHLY and YOUNG AMERICA. The price of the first is $3 per year; that of the latter is $1 per year. We supply the former, together with HALL'S JOURNAL, for the sum of $3.50; the latter, with HALL'S JOURNAL, for the sum of $2.25 ; and the three for the sum of $4.25. There are few families in America which might not receive these three magazines with pleasure and profit. We believe there are few which, having welcomed them for one year, would willingly part with them afterwards.

In connection with DEMOREST'S ILLUSTRATED MONTHLY, all subscribers receive, free, a copy of either of two celebrated chromos which they prefer —" The Old Oaken Bucket," or " The Captive Child." These are elegant works of art, and are fully worth the price asked for the magazines.

We will mail specimen copies of Demorest's publications and Hall's Journal of Health to all applicants at 10c each.

---

### HUMANITARIANS.

THE longer we live the more thoroughly are we convinced that there is a great deal more genuine humanity among those whose lives are apparently absorbed in business details than they get credit for. It may be that we are particularly fortunate in our acquaintances, but certain it is that a desire to do good penetrates the hearts of the most active and laborious workers into whose society we happen to be thrown.

This thought came to us anew on a recent visit which we made to the establishment of Madame Demorest, on Fourteenth Street. Of course everybody knows something of this lady as the leader of fashion, the originator of styles in ladies' clothing, as the inventor and manufacturer of numerous articles in ladies' wear, as the editor of a very prominent magazine for ladies, and another for young people. But probably few are aware that a high moral sense pervades the heart and controls the actions of this fashionable leader. It is something new and something pleasant to say, that this lady, to whom so many American ladies look for guidance in matters of dress, as well as in many other domestic affairs, is filled with an earnest desire to benefit, physically, mentally, and morally, such as are brought within the sphere of her influence. We were discussing the corset, and the injury which it had done to the flexible and yielding frames of young persons, and to our surprise we found that Madame Demorest was an enthusiast upon that topic. She had made a study of the female anatomy, and had devised a corset which should relieve, set off, and support the bust without compressing it or lessening in any degree the expansive power of the lungs. She called it her " Health Corset," and it appeared to be worthy of the name. To avoid the stoppage of circulation so commonly induced by garters, resulting in cold feet and congested vital organs, she provides stocking suspenders, which cost but a trifle, are quickly put on, and will, we are convinced, secure warm feet to many ladies who must surely suffer so long as they cut off the flow of blood by a close bandage below the knee. The principal vein of the leg sinks there beneath the muscles, and varicose veins and even palpitation of the heart may be induced by a tight garter.

To take from the hips and abdomen the necessity of supporting the weight of skirts with which ladies are clothed, this lady has devised a suspender which conveys to the shoulders the impending weight, and has the additional advantage of inclining, without forcing, the shoulders backward, thus giving added prominence to the chest, and imparting a very graceful *pose* to the body. We are impressed with the idea that Madame Demorest is a co-laborer with us in seeking to benefit humanity. Her field of usefulness is certainly a wide one. Her customers are found all over the land. Her magazines circulate enormously among intelligent and thoughtful people. Her words are read by millions every year. And we are glad to say that her teachings are ever on the side of temperance, good manners, good morals, and a pure life. She has done immeasurable good in the past; it is pleasant to know that her powers and opportunities for benefitting the human race are to-day greater than ever.

Madame Demorest has lately organized an immense tea store in connection with her business, and furnishes the purest and best teas to be obtained in China and Japan, where she has resident agents. In supplying the best articles in this line she is certainly doing great good.

---

### A COLOSSAL MESS.

We were walking down Broadway the other day, when our attention was arrested by the appearance, on the opposite side, of a number of men bearing aloft upon their hats some great red letters. For a moment we were puzzled to understand it. We thought of Hawthorne's poor woman, who stood with her scarlet letter in the market place, and wondered who had thus branded, by wholesale, respectable looking citizens of the metropolis. But not long were we kept in ignorance. As the column swung into something like line, we spelled out the word "Colosseum." We understood it all. It was the method taken by that shrewd manager, Mr. T. W. Kennard, to announce to the public that the attractive entertainment which he founded last year was about to be re-opened. For a moment we forgot the red-lettered men, and our minds reverted to the Atlantic and Great Western Railway, which Mr. Kennard built; to the lovely place at Glen Cove, once owned by Burton, the comedian, and afterwards transformed into the most beautiful of suburban villas by the taste and wealth of Mr. Kennard; of the great dinner which that hospitable gentleman gave to Sir Morton Peto; of his two beautiful steam yachts which cut the waters of the sound in splendor superior to any others afloat—all this, before we got back to his last great achievement, the Colosseum and its wonders.

Meanwhile our alphabetical advertisers were getting sadly demoralized. Several of the letters had stepped into an accommodating concern near by for —well, ice-water probably, leaving to our distracted vision only the last two syllables of the long word—"seum." A couple of stragglers then joined in, and, with the pride of Falstaff, took their places at the head, and we read "Lose-um." At a crossing, passing carriages mixed them up badly, and we had, in rapid succession, "lose," "muss," and "solo." Another change gave us "mess," which struck us as appropriate; but when the efforts of one of the party to reduce to a proper orthographic condition his men of letters resulted in nothing better than "cuss 'em," we were almost certain that they expressed the sentiment of the frantic commanding officer, and felt it to be our duty to hasten forward before the unfathomed resources of that colossal word should bring us still greater dismay. It was a "red-letter day" with us, and with all Broadway, and we walked off wondering how well-read men could blunder so, whatever might be the fact with the unlettered. But, in spite of these things, the Colosseum is likely to endure and to delight thousands with its charming novelties.

---

### THE GILDED AGE.

It was a genuine delight to sit in the cosy little Park Theatre on the night of the 16th of September and witness the first performance of the remarkable drama written by the gifted humorist, Mark Twain. It was a new and genuine sensation to see and comprehend a creation true to life, yet new to the stage, in the character of Colonel Mulberry Sellers.

For the first time in the history of the American stage, so far as we remember, we have a dramatic character which, while to be found nowhere else, is not a caricature; which, while indigenous to a locality, has no tinge of provincialism in word or manner; and which, while exhibiting traits and tendencies which might be deemed dangerous, is yet wholly void of offensive intent. To Mr. Raymond is due full credit for carrying into effect the designs which unquestionably pervaded the mind of the author. The actor admirably presented the large-hearted, broad-guaged, enthusiastic Missourian whom the author has so wonderfully created. The play and the part will live long after author and actor have passed away.

Our feeling for Colonel Sellers—a feeling in which, we think, every observer shared—was that we should be glad to take him by the hand, proud of his acquaintance, delighted to invite him to dine with us and to meet a few friends; not averse to hearing, with a little more particularity, exactly how he figures out the "millions in it," with which he announces each one of his vast schemes. He is eminently presentable—perhaps a trifle over-dressed as to the bosom, but then that style comports admirably with his freedom of limb and lofty and full-chested vigor; and we see at once that he could not be guilty of the smallest offence against decorum, and that meanness in him would be a thing impossible. When we compare him with other purely American creations of the dramatist, we see how utterly he eclipses them all. We think of Rip Van Winkle as a Dutch vagabond to be given a warm corner of the kitchen; and of Solon Shingle as an ignorant old booby who begs an onion which he is too mean to buy—who persistently seeks to extract the tough end of a clam from his teeth with his fingers; for whom, if you would entertain him, you must provide a table by himself, lest the proprieties should be offended and your company disgusted. You may be "taken in" by the whole-souled Colonel, but you cannot be offended by him. Your pocket-book may be lifted from its resting-place, but your "gorge" will not rise. Is it not likely, then, that a picture distinctively American, pervaded with a characteristic humor of which Mark Twain is the only living exponent—a humor which, however blunt, is never coarse—however extravagant, is yet founded on the clearest wordly wisdom—is it not only likely but certain that such a picture is destined to long life and large popularity ?

---

Arundel Tinted Spectacles.—We have been wearing for some days a pair of spectacles bearing the above name, and think they possess certain advantages over ordinary glasses. The matter is one of the first importance to all who are compelled to resort to these "helps to read," and therefore we await further trial before undertaking to speak positively concerning them.

### THE TARTAR WHO CAUGHT A TARTAR.

BY JOHN G. SAXE.

There's trouble in Hungary, now, alas !
There's trouble on every hand ;
For that terrible man,
The Tartar Khan,
Is ravaging over the land !

He is riding forth with his ugly men,
To rob, and ravish, and slay :
For deeds like those,
You may well suppose,
Are quite in the Tartar way.

And now he comes, that terrible chief,
To a mansion grand and old ;
And he peers about,
Within and without,
And what do his eyes behold ?

A thousand cattle in fold and field,
And sheep all over the plain,
And noble steeds,
Of rarest breeds,
And beautiful crops of grain.

But finer still is the hoarded wealth
That his ravished eyes behold,
In silver plate,
Of wondrous weight,
And jewels of pearl and gold !

A nobleman owns this fine estate,
And when the robber he sees,
'Tis not very queer,
He quakes with fear,
And trembles a bit in the knees !

He quakes in fear of his precious life,
And scarce suppressing a groan,
"Good Tartar," says he,
"Whatever you see
Be pleased to reckon your own !"

The Khan looked round in a leisurely [way,
As one who is puzzled to choose ;
When, cocking his ear,
He chanced to hear
The creak of feminine shoes !

The Tartar smiled a villainous smile,
When, like a lily in bloom,
A lady fair
With golden hair
Came gliding into the room !

The robber stared with amorous eyes ;
Was ever so winning a face ?
And long he gazed,
As one amazed
To see such beauty and grace !

A moment more, and the lawless man
Had seized his struggling prey,
Without remorse,
And—taking horse—
He bore the lady away !

"Now Heaven be praised," the nobleman [cried,

"For many a mercy to me !

I bow me still

Unto His will.

*God pity the Tartar !*" said he.

### CONCERNING HOTELS.

The finest hotel in the world, in the commanding chasteness of its structure, its aristocratic surroundings, the healthfulness of its situation, the elegance, commodiousness, convenience and comfort of its apartments, the quiet and order and harmony of its management, and the generous profusion of its table, is

THE WINDSOR, on Fifth Avenue, New York, half a mile from the Central Park; it has a frontage of one entire block of two hundred feet, on the finest street in the world; it is eight stories high above the basement, contains five hundred rooms, of which several hundred are chambers; its dining hall seats three hundred guests, and can accommodate five hundred and twenty-five persons at one time.

There is a tidiness and scrupulous cleanliness from cellar to attic, in store-room, kitchen, pantry, parlor and chamber, which is not to be equalled at home or abroad; it employs three hundred and seventy persons for its management, and its average expenses, including rental, amount to two thousand dollars a day. The proprietors, Messrs. Hawk & Weatherby, have had large and long experience in their calling, and for their capacity, management and integrity, command the respect of the business community of New York.

Not the least important personage in the management of any large hotel is the steward of the establishment, because to his care is committed all the table supplies, from a single egg to a quarter of beef. He is held to a strict accountability for every ounce of food which comes into the house; and to that end, he must see to it that nothing goes out of it which was intended for its use; hence, every one of its hundreds of servants must come in and go out at the same door, and no one of them can leave the place with a package an inch square, without presenting a ticket permitting the same.

At twelve o'clock on a certain day of each week the proprietors receive a report, showing what it cost to feed each guest, whether he had been there an hour or a week, and how much that guest paid.

A party had just left a private parlor, having ordered a basket of fruits, of which one half was left. "What becomes of that?" The waiter is required to take it to the storekeeper, who gives a receipt for it, which receipt must be accounted for.

Once a month every knife, spoon, fork, plate, cup and dish has to be counted; if one is missing, and is not accounted for, a particular person has to pay for it.

Twelve eggs came in to the steward, all were gone; the cook had sent only eleven into the dining-room. "Where is that other egg." It was decayed and could not be used. Without such a system of rigid accountability for everything, the bankruptcy of any large establishment would be speedy and certain.

Such is the division of labor, that every one knows his place and his business; hence there is no noise, no bustle, no hurrying here and there, no loud talking, no slamming of doors, no confusion. One man's time is wholly taken up in roasting or broiling beef; never touches any other kind of meat. One person has charge of all the fruit; another makes the pies; another bakes the bread; and so it is in every branch and department.

The charges for transient guests are five dollars a day; for permanent boarders and for families there are liberal deductions, varying according to the size, situation and number of rooms occupied.

There are two exemptions in connection with the "Windsor," perfectly delightful to think of. There is not a shop, or store, or "stand" anywhere to be seen; as far as the eye can reach, front and rear, there is nothing visible but aristocratic private mansions; and quite as comforting, for ladies especially, there is no crowd of men hanging around the doors impudently staring at every one who steps across the threshold, nor filling the corridors with boisterous talking, with the fumes of drink and tobacco spit, or the smoke of bad cigars; no loafing visitors or blacklegs, or well dressed burglars and thieves, watching around, "seeking whom they may devour;" no lounging about in chairs with feet high in air; no distressful beggars, no impertinent and persistent solicitors to purchase a tooth-pick, a subscription book, or a stolen hair-pin. In short, there is nothing at the aristocratic and noble WINDSOR to indicate that it is not the private mansion of a millionaire. May a large success await its courageous and enterprising proprietors.

---

### THE RELIGION OF HEALTH.

In several of our large cities there exists a somewhat costly luxury, which is extensively patronized by ladies and gentlemen whose means are sufficient to enable them to avail themselves of its advantages, but from the enjoyment of which the great masses are practically excluded. We allude to that system in the employment of which the human body is submitted to the influences of hot air, rubbing, percussion, washing in water of varying temperature, and cooling, and which for convenience, and not for the sake of accuracy, is called the Turkish Bath. It is a system which was unknown in Europe and America twenty years ago. No method of bathing resembling it in any marked degree has ever been known in Turkey. It is purely Italian in its origin, and has been but imperfectly imitated in Constantinople. It was the bath of the Romans when Rome was a power in the world. In Europe it was preceded by the vapor bath of Asia. Urquart, the ex-member of the British Parliament, and the life-long foe of Lord Palmerston, introduced the Roman system into England, and termed it the "Turkish Bath." Here it speedily superseded the "lamp bath," a portable system not unlike the "rum sweat" or alcohol bath, formerly and still practiced by old nurses among us. This lamp bath, before Urquart's innovation, was the only means known for the application of dry hot air to the human frame. Heat had for some time been professionally employed in the form of hot water, at the celebrated water-cures of Drs. Wilson and Gully at Malvern, Dr. Smith at Ilkley-Wells, Dr. McLeod at Ben Ridden, and Dr. Lane at Beulah Spar.

About the year 1855 the Hon. Mr. Urquart returned from a visit to Rome, impressed with the value of the hot-air bath as a preventive of disease and as a curative agent, and began to make known in a public manner his convictions of its importance. A severe attack of malarious fever, of a type frequently fatal among unacclimated Roman residents, had been warded off in his own case by the hot-air bath, unaccompanied by other treatment; while friends about him, who had employed the more "regular" medical means, had either languished long or speedily succumbed. Naturally, therefore, the convert to this phase of Romanism, owing, as he felt certain, his salvation to the system, became its most earnest advocate.

At this period he made the acquaintance of one Dr. Barter, whose name has since become famous all over Great Britain. A believer in the sanitary influence of heat, it was not difficult for the enthusiastic Urquart to saturate him with visions of future good to be accomplished and fame to be won by the introduction of the Roman baths into his native land.

With Dr. Barter to plan was to execute, and the result was, the establishment, in 1856, at St. Ann's, near Cork, Ireland, of the first hot-air bathing place in the Western World. Here was established the misnomer which has since followed it, for it was called the Turkish Bath. The building still stands, although no longer used for baths; improvements in the system having rendered its primitive arrangements obsolete. The construction of a building in which the human body should be immersed in pure, dry air, heated to a high temperature, yet constantly renewed, was not at that period deemed practicable, either by architects or by medical men. One architect replied, when consulted by Dr. Barter, "Why, sir, if you let fresh air in freely, you will be, in effect, heating the whole county." So the Doctor allowed them to make him a hot closet, without ventilation; and, to "purify the air," as it was then termed, he placed pans of water in the furnace-chamber to give off vapor. The result was that he found himself provided with a simple vapor bath. Discovering this when too late, and being fully impressed with the idea that the best effects of heat in treating disease could only be secured by the use of heated air constantly renewed and consequently devoid of moisture, he devoted much time and thought to the work of devising an edifice in which what he considered the true theory of hot bathing could be efficiently carried out. This labor finally resulted in the erection of his great establishment at "St. Ann's-on-the-Hill," followed speedily by elaborate bathing-houses on the same plan at Killarney, Bray, Belfast, and Dublin. The edifice erected at Dublin far excelled all the others in point of completeness and splendor, and to it was given the name of "The Hammam." Dr. Barter also endowed a similar bathing-house in London, in 1859, to the extent of $10,000. To Ireland, then, is the civilized world indebted for the importation, from the home of the Pope, of that system falsely called "Turkish," which stands perhaps without a rival as a remedial agent in diseased conditions, and as a luxury in health. While in the case of Urquart, conversion was the result of what he deemed a miracle wrought upon himself, in the case of Barter the conversion came first, through the contagion of that enthusiasm with which Urquart was filled. Barter was an educated Irish physician, of the ancient, Allopathic type. His Irish nature, open to ready conviction on any subject which his reason approved, seized the thought of Urquart almost before it was uttered, and he hastened to reduce it to practice. It only remained for his conversion to be enforced by future observations. That enforcement was not long delayed. He had been a martyr for years to dyspepsia and various distressing stomachic complications, and from all the worst features of his complaint he gained speedy relief in hot-air baths. He found in the manipulation accompanying the sweating process a healing and invigorating agent, which, for a long time, defied his powers of comprehension. This remained to be at once developed and explained by American disciples of the new school.

Of course the United States could not long remain unsupplied with a system which had won so speedy and great a renown in the best circles of Great Britain. In 1860 Dr. Esterbrook opened a small bath in Boston, followed by Shephard in Brooklyn, in 1861; by the establishment of Dr. E. P. Miller, in Laight street, New York, in 1862, and by Lackey in Chicago, Hanscom in Milwaukee, Adams in St. Louis, and Wilson in Philadelphia.

Of all Americans, Dr. Miller has entered into the work with the greatest vigor and enthusiasm. He has from the beginning been filled with the hope and belief that at some period not far distant, hot air would be nearly as plentiful and as free as cold air. His labors have been largely directed to the solution of the problem—How can the great benefits of this system be extended to all?

In opening the Laight street house, he sought to combine all the excellencies of the system as practiced by Dr. Barter. He visited Europe and carefully examined whatever was to be seen in the line of his specialty. At all points he was aided by the enthusiastic Irishman. Complete drawings were furnished him, and he returned to the United States, fortified with all the wisdom then extant in other lands.

When organized, the Laight street place was excellent of its kind. But the restless spirit of its founder could not remain contented. He must have new improvements, greater space. He must have a higher locality, better air and increased facilities. So he bought three houses on Twenty-sixth street and united them in one establishment. All the improvements which experience could suggest were added here. The great heating apparatus was so constructed as to secure any amount of pure air, constantly replenished and raised to whatever temperature might be found desirable. Added to all this, water in copious abundance—hot, cold and tepid—was supplied. Rooms for the employment of electricity also found a place in the new institution. To secure for such as should come as patients, wholesome food, a hundred rooms were fitted up for boarders.

"Miller's Bath Hotel" is now in the fourth year of its existence. It is one of the pleasantest houses which can well be imagined. It is not a hospital for the sick, but a delightful resort for all. It is patronized by the thoughtful, the intelligent and the refined. Professional people abound here. The great parlor is a blaze of light each evening, and music and innocent amusement and recreation abound.

Of course, with a progressive nature like Dr. Miller's, it would not be consistent for him to remain contented with his past achievements. He is convinced that when the benefits of this hot-air system are fully understood, legislatures and congresses will deem the establishment of these health institutions as important as the support and endowment of schools and colleges. He entertains a hope that by-and-by the cost of a hot-air bath, with the manipulation therewith connected, will be supplied for a very small sum—will be, in effect, free. He has called in consultation, with a view to the accomplishment of this end, the most ardent apostles of this new and cleanly faith. In doing so, he could not, of course, pass by the foremost advocate of the system—the hero of a hundred bloodless battles among Fenians and Communists—the robust survivor of we know not how many Turkish Baths—Mr. George Francis Train. To him Dr. Miller has communicated his plan for the organization of a company, to be called the Religion of Health Company, with a capital of $10,000,000, for the establishment of these institutions in all the chief cities and towns in the United States, and asks his aid. Mr. Train responds to Dr. Miller's suggestion by offering to give freely the whole of his fortune for the advancement of the cause, provided Dr. Miller will do the same, and will devote the remainder of his life to the work of constructing and superintending these hygienic institutions, without other charge than his own support. That some practical results will be evolved from the united efforts of these two earnest evangels of the new dispensation, is altogether probable; and it would not surprise us if the great public were shortly called upon to aid in some comprehensive scheme for the construction of places similar to the famous one on Twenty-sixth street.

That their general introduction would accomplish great good, we are prepared to believe. We know that the skin is the great system of sewerage through which the body throws off its waste and its poisons. To dam up the sewer is death; to open it, when partially obstructed, is a necessity to healthy action. This and much more the so-called Turkish Bath does. It supplies hot air, dry air, pure air. The dry heat destroys the disease-germs, and the perspiration evolves them from the system. The water drunk dilutes the excretions, and the body is thus washed out. The kneading, rubbing and manipulation encourage and facilitate the exit of impurities, and thus disease is eliminated, and only the sound tissues remain.

This "Bath Hotel" is appropriately named. Its patrons are not compelled to bathe, but they incline to. For less than the cost of living at any other equally well-conducted, first-class hotel, individuals can board here, occupy well-appointed rooms, and enjoy the baths. If suffering from any ailment they are treated in such a manner as their condition demands. The hot-air bath and its adjuncts become, under Dr. Miller's guidance, the balm for every ill, the most powerful and successful among remedial agents. The sufferer, weakened by disease, has constant and tender care. Two baths a week may be better for him than the two baths a day which are prescribed for the chronic patient. For one, a half hour in the hot room may be ample; for another two hours may not be too much. It is a matter of knowledge, experience, practice; and all these the patient has the benefit of. The name of "Resurrection Bath" has been given to this system by a patient who was raised from death's door under Dr. Miller's hands. There are not wanting enthusiasts who believe, from the results experienced in their own cases, that if a person is not more than two-thirds dead he can here be restored to health and vigor.

As to the safety of the hot-air bath, there should be but one opinion, and that a favorable one. The superintendent of Dr. Miller's baths has administered over 70,000, and has never known injury to result when the directions have been followed. Can the same be said of any other system?

---

### WOMAN AS A PHYSICIAN.

There is no reason in the world why worthy women should not be medically educated, and there are a thousand reasons why they should be. They are and have ever been the only nurses deserving the name, and it is folly to say that the deft hands and sympathetic hearts which constitute the most effective attendants in times of sickness cannot be educated to such a degree as to make them the best judges of the proper medicine and treatment to be employed. It may not be that the female sex will ever wholly supersede the male as doctors, but we are prepared to believe that such a transformation could occur without imperiling human life or leaving any serious void in any direction. Woman has shown herself capable, as often as tested, of performing all the functions of the physician and surgeon; and now that she has discovered her power and capacity, she has the good sense and the strength of character to compel for herself whatever advantages are enjoyed by her brother man, not only in the direction of education but also in the line of that fame and profit which follow hard upon it.

We heartily encourage all the efforts which our sisters are making to secure for themselves independence and power in all possible directions, and in none more than in their persistent endeavors to secure the means of acquiring a thorough medical and surgical education for the earnest and intelligent of their own sex. It is abominable to reflect how long medical universities have been closed upon refined females, while freely open to all classes of men who can pay the fees. Any one who has ever attended a medical college in a large city, and has watched the great majority of the students, with their rude manners and their vulgarity, will be ready to admit, unless a prejudiced witness, that some leaven of that politeness and decency which most human beings manifest in the presence of the gentler and purer sex, is greatly to be desired, and that the admission of ladies to the classes would, perforce, impart a useful lesson to the many men of low instincts who get mixed up with the more refined in our modern medical schools.

When we see a woman strong enough and consistent enough to seek to do anything and to do it well and persistently, we are charmed, and we feel the strongest impulse to reach out our hand cordially and to bid her an earnest God-speed. We do not find a great many of these. Nearly all appear to lack continuity, fixedness of purpose. They put their hand to the plough, and look back. This is, no doubt, because they are timid. They do not have before them, and on all sides, the multitude of monuments of splendid achievements which sustain and encourage men. They see few women pushing out and winning fame and fortune. But all this is changing. Fifty years hence, struggling girls, longing to do something and to be somebody, will be able to point to thousands of women who have striven, who have prospered; who have risen to a distinction unexcelled by any man living.

So let women press forward and faint not. Let them plant, broad and deep, the structure of a good education. Let them declare that what man has done as a healer, they can do, and far more. They can say this honestly, for the art of healing is only just emerging from barbarism ; a thousand old errors engrafted by Galen and Hypocrates and Paracelsus still exist, and are to be eliminated by intelligent women or intelligent men.

There are some notable examples of success in medical practice among women. Take the case of Mrs. Clemence S. Lozier, who resides at No. 361 West Thirty-fourth street, New York. She has been a physician and a teacher for more than forty years. She was, to be sure, born of a race of doctors, but she entered upon her life-work as physician and lecturer almost alone. No college was open to receive her and confer upon her a degree entitling her to practice. Many years passed before she could secure a degree. See how things have changed! She is now Dean and Professor of a popular and successful medical college, having its Faculty, consisting of fifteen able and learned men and women, granting to all women, young and old, the privilege of a thorough medical and surgical education, as complete as any man can secure, and all at the cost, we believe, of about $200 !

We want to appeal to fathers and mothers who have daughters growing up, and to ask them if it is not their duty to secure for them a clearer knowledge of anatomy, physiology and the effects of medicines in health and in disease, than is taught in the schools. We wish to impress upon parents the absolute necessity which exists for medical education for women. We wish to assert, in the strongest terms, our belief that only through the education of our daughters is the human race to be redeemed from its heritage of disease and premature death.

We are in full sympathy with Dr. Lozier in her great work. We have long recognized her as the leading evangel in the noble mission of education for woman. We rejoice that the efforts which she has so intelligently and persistently made in this direction are resulting so successfully. We trust she may long be spared to a community and a nation which she has so signally blessed by her earnest efforts and her noble example.

One word more. We are safe in saying that " The New York Medical College and Hospital for Women," at the head of which Dr. Lozier stands, is one of the best schools open in the world for the medical education of females. Its new term begins on the 13th of October. Before that date the JOURNAL will be in the hands of its many readers. Will not *some* among the intelligent parents who read these words, consider seriously the call which we here make upon them to present from among the daughters who are their joy and pride, at least one who shall become a benefactor to her suffering race, and whom multitudes shall rise up and bless ? They will find in Dr. Lozier a friend and counselor to be trusted and revered.

---

JAMES MILLER & Co.—This well-known house, located at 647 Broadway, will receive subscriptions for HALL'S JOURNAL OF HEALTH, and receipt for the same.

### TASTELESS MEDICINES.

It is evident that medicine will be taken just as long as our worn out bodies demand remedial agents. That the sick will forever fly to the doctor and the druggist, for relief from all the ills that flesh is heir to, is clear; and it is equally apparent that it is the duty of the medical man, not only to refine and purify and concentrate the drugs employed, but also to eliminate from them the nauseating and unpleasant conditions by which the use of the larger part of medical preparations is attended.

We hail with pleasure all intelligent efforts which we observe in this direction, and shall rejoice most heartily in any discovery which will deprive the medicines exhibited, of their offensive flavor. To this end it has been common of late years to coat the surface of pills with a sugar compound, by the aid of which they are swallowed without exciting emotions of disgust. But only solid substances can be readily formed into pills. There are many fluid medicines of great thereputic value, but unpleasant to the taste, and these must either be swallowed in their natural form —to the infinite annoyance of the palate— or must be partially disguised by the addition of some more palatable substance; or must be conveyed into the stomach by the means of some tasteless vehicle. Against the use of a large class of unprotected fluid medicines, the sense of taste utters its strong protest; so strong, indeed, that the stomach is very apt to join with it in condemning the aggression. This is the most serious obstacle met with by physicians—the reluctance of the stomach to receive and retain the medicines swallowed. If the sense of taste could be shorn of its power during the administration of remedial. agents, "irritable stomachs" would be far less common, and the best effects of the medicines employed would be more frequently secured.

We should be rejoiced if the use of all drugs could cease at once and forever. It would delight us to know that in the future all diseases were to be successfully treated without medicines. But that happy millenium has not yet reached us. We must still swallow our unpleasant potions, and fight fire with fire. Let us, then, seek to render the means which we believe it to be our duty to employ, as endurable as possible.

An expert chemist, Dundas Dick, of this city, is accomplishing great good in this direction. His method of administering unpleasant fluids is to put them up in little bottles for the use of physicians and the public, and to cause the patient to swallow bottle and all! Our readers may think this a singular statement, but it is strictly true. The only explanation requisite to a clear understanding of the case is as to the nature of the bottle. The fact is, the bottle is made of pure gelatine—such as jellies are made of—and while it affords a perfect protection to the confined medicine, it is almost instantly dissolved in the stomach. The full effect of the medicine is thus secured without unpleasantness, while the vehicle or vessel used, simply acts as demulcent and useful food.

Dr. Dick confines within these small, gelatinous cells several kinds of medicines which are largely employed, and which are difficult of administration in their naked condition. These are castor oil, cod-liver oil, oil of sandal-wood, the balsams, &c., and the protection thus afforded the palate is securing for these potent drugs a very large sale.

Of course it would be a very easy thing for a chemist who lacked conscience and was devoid of principle, to deceive the consumers of his articles. It is known that very impure and wretched drugs have been put up in this form by the unprincipled, and that the use of the capsule covering has thus been brought into great disrepute. To heighten the reputation of this admirable vehicle is the work of Dr. Dick, and he accomplishes it by the strictest fidelity in the preparation of his goods. He secures only pure articles, regardless of cost, and puts them up with scrupulous care. His prices are necessarily higher than those of the cheap and obscure vendor, but they leave for him only a small margin of profit, because of the superior excellence of his goods.

In nothing is more care necessary than in the selection of the articles swallowed. If this is true in the matter of food used by the healthy, how much more important is this caution in that of the medicines taken by the sick! The reputation of the remedies of which we have spoken rest entirely upon their purity, and leading physicians testify to their trustworthy qualities. So apparent is this to medical men in large practice that they are careful to prescribe no capsules except these, for the purity of the contents of which they can personally vouch. So far as we have been able to learn, Dr. Dick's products stand at the head of all articles in his line. That they are precisely what they purport to be is, we believe, universally admitted.

It is curious to observe the manufacture of these tasteless preparations. Every capsule passes through not less than twenty hands, and is subjected to not less than five critical examinations. They are then carefully wrapped in tin-foil and neatly boxed for transportation. The industry is a rapidly growing one, and new articles of recognized merit are being thus protected by the film of harmless gelatine. The articles thus put up are solely such as are employed by the educated medical man, and no quack compounds or secret preparations are found in his catalogues. The vehicle of gelatine is employed, not for purposes of concealment or disguise, but simply to secure for the remedies an easy passage to the stomach. Each article used has its place in medical science, and none has more rapidly attained to a position of prominence than the oil of sandal wood, which is found to be superior, in the diseases to which it is adapted, to all other remedies. Castor oil, generally so unpleasant to the taste, is readily swallowed in its delicate envelope, and its effects are in no degree impaired.

We are convinced we are doing a real service to those who imagine themselves compelled to swallow nauseating medicines, when we call their attention to these elegant preparations of Dr. Dick. If we were able to convince them that they could get along without drugs in many cases in which they deem them of the first importance, we should most heartily congratulate them and ourselves. But, since this cannot be, let us urge them not to unnecessarily offend the palate, with which the stomach is so sure to sympathise. The tasteless medicines prepared by Dr. Dick are for sale by all good Druggists, and may be bought in quantity of the general agent, Victor E. Mauger, No. 110 Reade street, New York.

---

### A METROPOLITAN LANDMARK.

There are landmarks in this great city, not less among the firms which have come up from small beginnings to positions of prominence and importance, than among the edifices which they occupy. Indeed, the social and business position acquired by long years of integrity, is a far more imposing and enduring monument to perpetuate the name and fame of our leading business men, than the costly palaces in which they abide. The demands of commerce and the ever varying tendencies of trade, may force our merchants from one locality to another; from one costly building to another still more costly; but the reputation acquired by years of earnest and honest labor never varies, and is never lost. External surroundings may change; the good name is unalterable and changeless. That poet of the English people, who declared that

> "Pure hearts are more than coronets,
> And simple faith than Norman blood,"

has beautifully uttered this sublime truth—that the truly great are the truly honorable. We can point this moral better, perhaps, by an appropriate illustration, than in any other way.

In 1810, when New York was a small city, its chief jewelry store was located at 166 Broadway. This was then considered pretty well "up-town," a good ways, in fact, above the principal residence portion. Several blocks below, Mr. John Jacob Astor resided, at 55 Broadway, and the buildings north of St. Paul's church were very few. At No. 166 Broadway, then, this jewelry establishment began, in a small way, and there it continued with few competitors and without an absolute rival for twenty-three years. In 1833, larger accommodations were demanded, and the house sought them by moving across the street and a few doors further up. For fifteen years its constantly increasing trade was conducted at 181 Broadway, and then, in 1848, it was found necessary to push onward with the advancing tide, and secure added facilities still further to the north, at 247 Broadway. For twelve years this fine store chiefly supplied the demands of the growing city, until another change became necessary. In 1860 that change was made. This time it was to a splendid edifice erected by themselves at 565 and 567 Broadway, nearly opposite the Metropolitan Hotel.

To the present generation the house of which we speak is known as Ball, Black & Co., under which name its greatest achievements have been accomplished. It was this firm which erected, on the site alluded to, the first absolutely fire-proof building in New York, and they constructed beneath the great building the first vault for the use of the public, thus practically inaugurating the safe deposit business of this country.

Amid all the changes of sixty-four years, this substantial house has steadily pursued the even tenor of its honorable career. The name and style of the firm has undergone modifications, as partners have died, or have retired with a competency, but the ancient leaven has still remained to leaven the whole lump. It has been a leaven of integrity and fair dealing. The plan adopted away back in the beginning of the century was to supply the best of everything, at a moderate profit, and to guarantee its excellence. Of course, such a plan must succeed, and there can be no more marked evidence of that success than the history of this house affords. During all these years they have stood at the head.

A recent change in the *personel* of the firm brings into prominence some of the younger members. It is to be known hereafter as Black, Starr & Frost. These gentlemen have grown up with the establishment, and have long been important parts of the great whole. The method and system which have ever pervaded its business still obtains. The old principles of honor actuate one and all. The ancient prestige of the house will be watchfully sustained by the gentlemen in whose hands are now its destinies. It is their *alma mater*, and its honor is precious to them. Indeed, it has been the great university from which the ranks of the trade in this particular line, all over the country, have been recruited. You will find numerous graduates from this house at Tiffany's and at all the best jewelry establishments throughout the land. To be educated here is deemed recommendation enough, not only in the line of business, but in the more important direction of honesty and fair dealing.

It is interesting to observe the careful method which exists in the conduct of this great business. The stock is immense, and is, of course, made up of the most precious things in gold and silver, and jewels and works of art. Were it not for a system of checks and balances, extending to the most insignificant articles, great losses might occur and long remain unnoticed. Accordingly, a strict record is kept of everything, and that record is changed the moment the article is disposed of. The whole stock is carefully examined frequently, and serious losses by theft or by carelessness are rendered nearly impossible. No loss, however small, can occur without speedy detection.

The purity and integrity of the goods are proverbial. They will not offer for sale a fictitious article. They will not sell you a watch which will not serve you well. You cannot purchase at this house what purports to be fine silver ware, and find it reduced in value by alloys. The result is, that what you obtain here will be prized by you as long as you live. You may feel, when you make a present from this stock, that your reputation is safe with the recipient of your bounty, because the gift will be the best and choicest of its kind.

It is a pleasure to deal with these worthy gentlemen. It is folly to suppose, as many do, that because their great marble building is palatial—because its interior is imposing and grand—that the prices must be out of reason in order to support such grandeur. The truth is, there is no safety in buying a class of goods which may be counterfeited as readily as can articles in gold and silver, of any but a house of high standing. It is poor economy to seek out obscure and cheap localities for the purchase of articles in the fabrication of which fraud is not only possible but is constantly practiced. You may rest assured that the cheap article of the obscure dealer is certain to prove unsatisfactory and costly in the end.

It may be that the inexorable demands of fashion will shortly compel this ancient and honorable house to push still further up into the residence portion of the great city, which has been built up, as it were, under their very eyes. Be this as it may, it is certain that a host of the best citizens will follow them with their patronage, and that their thousands of friends in all parts of the country will be sure to find them out. The house will still remain a landmark of honor and integrity in the vast metropolis.

---

### AMERICAN ART.

We are inclined to the opinion that the American people are destined to reach the proud distinction of being the most artistic people in the world. While they do not live in the dreamy and sensuous atmosphere of those nations which have attained to the highest distinctions in art matters, they yet manifest their artistic taste in a thousand rational ways, and especially in the method with which they seek to adorn and beautify the useful objects of life. With them the *utile dulce* is certain to be blended. But, until recently, we have supposed that in the matter of bronzes we must look to the products of France for elegance in design and artistic merit. Our opinion has been essentially modified by a visit to the spacious warerooms of Messrs. Mitchell, Vance & Co., 597 Broadway. This house is the leading one in America in the manufacture of bronzes, clocks, chandeliers in gilt, bronze and glass, and in all varieties of gas fixtures.

The building in which these goods are displayed is a grand receptacle of art. Its entire seven stores are devoted to the business. Here you may see works of art in bronze, rivaling, if not excelling, in taste and exquisite workmanship, any of the productions of foreign artists, ancient or modern. The several floors are filled with beautiful and useful goods in metal and glass. It will take hours to see and comprehend all the wonders of this marvelous display.

Your eye lights upon an exquisite mantle ornament in bronze, which proves to be a figure of Mercury. In form and feature and attitude you discover grace itself. You recognise in the posture of this "winged herald of the dawn" the description of Shakspeare's "Mercury, new-lighted on a heaven-kissing hill," and you are free to admit that none but a genuine artist could have conceived, none but a true artizan could have executed, a work of such infinite excellence.

You are apt to think that the production of one such beautiful figure in enduring bronze would be sufficient to immortalize its makers. But as you follow with your eye the thousands of remarkable designs in figures, urns, vases, pitchers, clocks, statues, groups, animals, card receivers and stands, thermometers, candlesticks, jewel boxes, the various classical and mythological subjects which art has sought to exemplify, and the multitude of graceful things which so adorn the homes of the refined, you are filled with wonder, and you find it difficult to understand how one house has been able to unite in itself the talent and capital and taste requisite to produce all this bewildering maze of beauty. You will, perhaps, take it for granted that the art-centres of the old world have contributed to this display. But you will learn that not only does this house import no manufactured goods, but also that it purchases none, depending entirely upon the resources of its own vast factory on the corner of Twenty-fourth street and Tenth avenue, for its products, whether they be of glass or metal; and, further, that nearly all the designs employed by them originate with themselves.

In the line of gas-chandeliers of original design, the public edifices and many of the sumptuous residences of New York attest the skill and taste of this house. One of their greatest works will soon be exhibited in the new Masonic Temple on the corner of Sixth avenue and Twenty-third street. The gas-fixtures here are entirely original, and were designed by the manufacturers. They represent in bronze the five orders of architecture, and are probably the most elaborate of their kind in this country.

Perhaps we may venture to remind our readers that there are advantages in purchasing goods of those who manufacture them. It may be that the prices do not differ greatly, but it is certain that the customers have a larger stock from which to select if they buy of first hands, and are more likely to be satisfied. They feel that if they fail to find what they desire, it will be useless to look in other quarters.

It is a satisfaction to learn, as we do, from this house, that their long labors in this field of enterprise have been signally rewarded in the improved taste of the public. This is shown by the growing disposition of buyers to select with increased care such gas-fixtures as are suited to the improved architecture and decoration of their dwellings. It could hardly be otherwise. The taste of the patron is certain to be educated by the manufacturer. The consumer may be content to worry along with the old styles so long as nothing better is afforded him. But if the energy and skill of the artizan are exerted to produce new and improved styles, more beautiful designs, shapes more graceful and artistic, the public are sure to follow speedily along the pathway illuminated by his genius.

Growth in this direction of useful art is cumulative. The circle of influence exerted by a single work of beauty may be vastly beyond the expectation of the designer. The thing of beauty is a joy forever. The good seed sown by Messrs. Mitchell, Vance & Co., in times of prosperity has borne fruit even during the season of depression. Their great factory has never stopped, nor has their working force been materially diminished. They have looked upon the period of stagnation as but temporary, and have employed the year in active preparation for the good time coming. They anticipate years of business prosperity for the country, and in this faith they are earnestly striving to excel all their past efforts.

---

Crown Perfumes.—The odorous extracts bearing this name are superior, in point of delicacy, to any within our knowledge, and are rapidly coming into general use. The bottles are unique, the upper portion of the glass stopper being in the shape of a crown. This gives it an ornamental appearance and forms a most expressive trade-mark for the goods. They are destined to acquire immense popularity.

THE HOUSE OF STEINWAY.

WE shall commence next month, a series of articles descriptive of this great house, with historical allusions to its founder. The articles will, also, describe the different methods of constructing pianos, and will, we hope, prove a valuable addition to the literature of this great branch of art-industry.

---

VAN DEREN'S REMEDY FOR CHOLERA INFANTUM.—For a few months past we have published the advertisement of this remedy. The proprietors have not advertised it elsewhere. Its sales have, therefore, been chiefly to readers of the JOURNAL OF HEALTH. We hear the best accounts from its use. Several mothers have written us to say that its action is perfect. We have ourselves given it in some very bad cases of dysentery in children, and always with gratifying results. We know of what it is compounded, and can vouch for its safety. The little ones take it as readily as sugar plums, and it is delightful to see them rally under its influence. It should be in every house where there are children. Hall & Ruckel, 218 Greenwich street, New York, are the agents.

---

SUBSCRIPTIONS TO THE JOURNAL.

MR. F. EDWARDS, the well-known boot and shoe manufacturer, 166 and 168 Atlantic avenue, Brooklyn, suggests to us, that he can secure a good many subscribers to HALL'S JOURNAL OF HEALTH. We have told him to take all that come, and to issue receipts for the money—$2 per year, or $1 for six months.

Mr. Joel McComber, the inventor of the very valuable improvement in boots and shoes, will also take subscriptions at his office, 21½ Spruce street, New York.

---

MME. DEMOREST will receive subscriptions for HALL'S JOURNAL OF HEALTH, at her fine place on 14th street. In fact, nearly all the best publishers and booksellers in New York and Brooklyn desire to keep the JOURNAL on their counters, and to receive subscriptions for it. In this way our list is rapidly increasing.

NEW HOMES; OR, WHERE TO SETTLE —12mo., cloth binding; 100 pages, with map.

Being a full description of the area, population, topography, soil, climate, products, cost of labor, cost of farm animals, price of living, wages of labor, &c., in seventeen of the Western States and Territories.

To which are added new and valuable articles in relation to cattle and sheep-raising, formation of farms, fruit culture, &c.

This new and elegant volume will be mailed to subscribers, free of postage, on receipt of publisher's price—$1.

E. H. GIBBS & Co., 84 Broadway.

A copy of this elegant book and HALL'S JOURNAL OF HEALTH, for one year, will be sent on receipt of $2.50.

---

THE twenty-first volume of HALL'S JOURNAL OF HEALTH AND MISCELLANY—being for the year 1874—will be bound and for sale before November 15th. The price will be $2.50, at the office of publication. When sent by mail the amount of postage—30 cents—must be sent to us.

E. H. GIBBS & Co., 84 Broadway, N. Y.

---

APPLETON'S EUROPEAN GUIDE BOOK. Morocco, gilt-edges. 750 pages. 20 maps and 150 engravings. Being a complete guide to England, Ireland, Scotland, France, Germany, Holland, Switzerland, Italy, Austria, Russia, Norway. Denmark and Sweden.

It contains complete directions for reaching any point in Europe, the time occupied and the expense, and the name of all the recommendable hotels.

It is the best compendium in existence of the history of Europe, its topography architecture, works of art, etc.

The publishers of Hall's Journal of Health and Miscellany, will send this valuable book, post paid, on receipt of publishers' price, $6.00 ; or the Guide Book and Hall's Journal of Health for one year, will be sent for $7.50.

E. H. GIBBS & Co.,
Publishers Hall's Journal of Health.

## Figures and Tables

**Fig. 1. f1:**
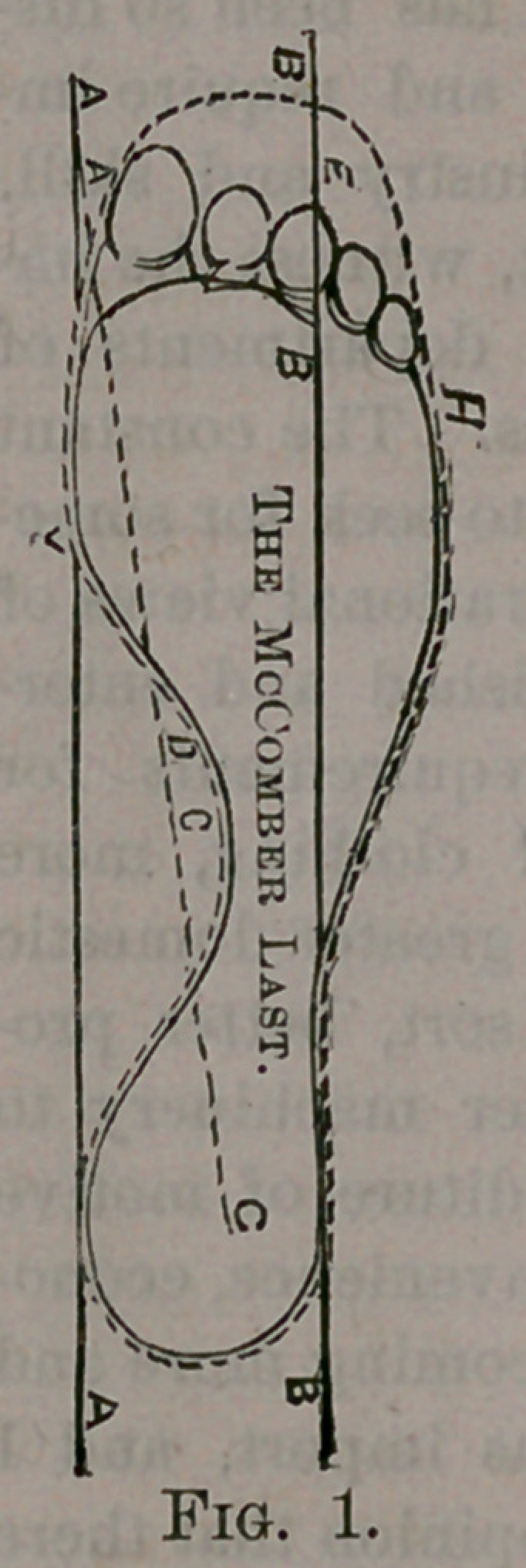


**Fig. 2. f2:**
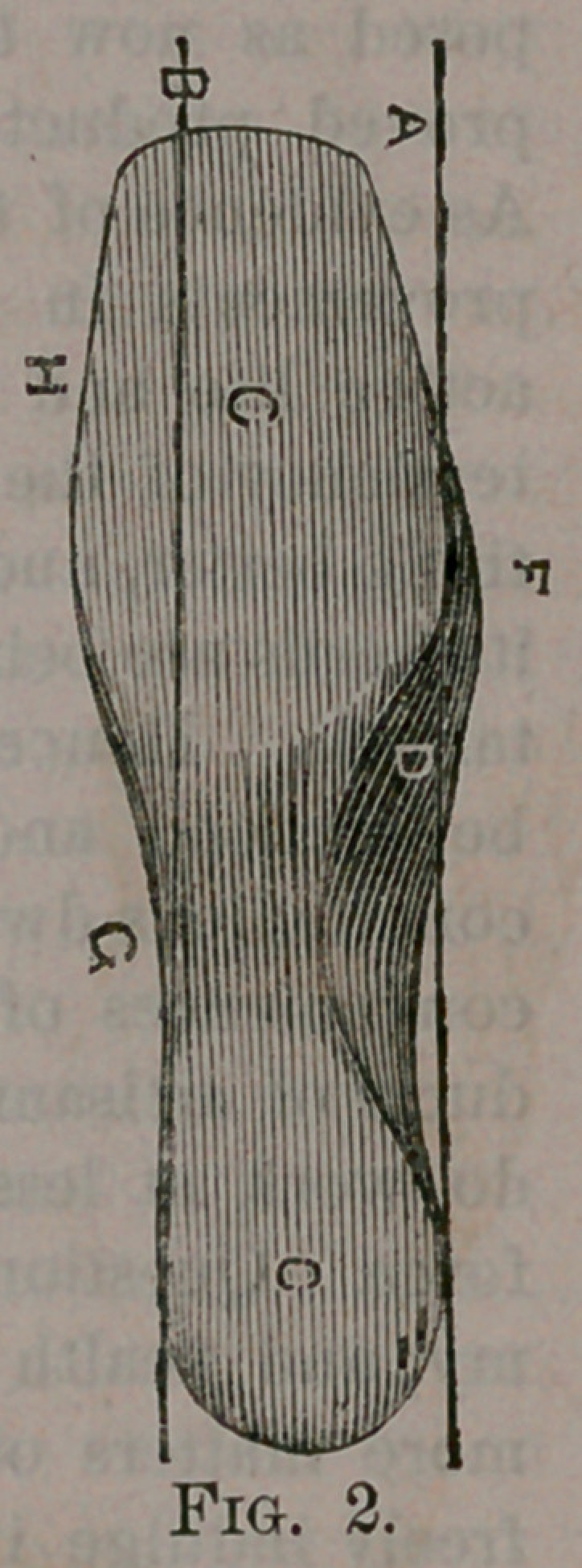


**Fig. 3. f3:**
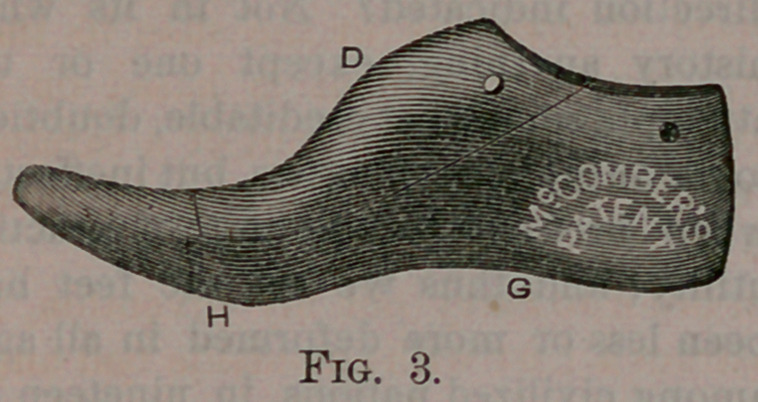


**Fig. 4. f4:**
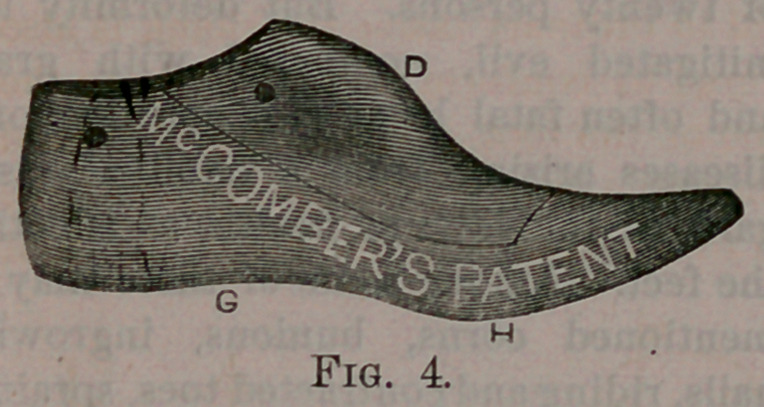


**Figure f5:**